# Estimating the Impact on Parents’ Infant Feeding Choices of Increasing Age Guidance and Adding Sugar Warning Labels to Commercial Infant Foods: A Mixed-Methods Study

**DOI:** 10.1016/j.cdnut.2025.107443

**Published:** 2025-04-15

**Authors:** Rana E Conway, Ivonne Derks, Florence Sheen, Andrew Steptoe, Clare H Llewellyn

**Affiliations:** 1 Research Department of Behavioural Science and Health, University College London, London, United Kingdom; 2 School of Sport, Exercise, and Health Sciences, National Centre for Sport and Exercise Medicine, Loughborough University, Loughborough, United Kingdom

**Keywords:** parents, food policy, infant foods, weaning, food labeling

## Abstract

**Background:**

Minimum age guidance on commercial infant food (CIF) does not always align with government advice. Despite marketing suggesting that CIF is healthy, many are high in sugar.

**Objectives:**

To estimate the impact on parents’ feeding choices of increasing age guidance on CIF and adding sugar warning labels (SWLs), including differential impact according to socioeconomic position.

**Methods:**

A convergent parallel mixed-methods study was conducted with parents or main caregivers of children aged 6–23-mo in the United Kingdom. In an online quantitative survey, participants (*n* = 1237) reported feeding behaviors and were asked about CIF choices when randomly assigned to view products with different ages or sugar labeling. In qualitative focus group interviews, parents (*n* = 22) discussed feeding choices and labeling. Quantitative results were analyzed using χ^2^ tests and binary logistic regression, while framework analysis was applied to qualitative data. Quantitative and qualitative findings were then integrated for overall interpretation.

**Results:**

Similar numbers of participants selected products labeled “6-mo+” (84.5%) or “4-mo+” (88.0%, *P* = 0.07), but fewer chose snacks labeled “12-mo+” (86.3%) compared with “6-mo+” (96.6%, *P* < 0.001). In focus groups, parents described multiple factors guiding their feeding choices; they felt labels were misleading and wanted age guidance on labels to align with government recommendations. Both survey and focus group participants trusted that CIF was low in sugar. CIF desserts displaying SWLs were chosen by 27.1% fewer participants than CIF without SWLs (*P* < 0.001). Differences according to label condition were similar, irrespective of income, occupation, or education. Focus group interviewees strongly supported the introduction of SWLs on CIF.

**Conclusions:**

Results suggest that the mandatory use of minimum age guidance and SWLs on CIF could provide parents with agency and shift feeding behaviors toward closer alignment with recommendations. Importantly, the impact of these label changes appears socioeconomically equitable, and they are supported by parents.

## Introduction

Good nutrition in the early years is essential for healthy growth and development [1]. However, by the time children in England start primary school (aged 4–5 y), 1 in 5 is already living with overweight or obesity [2]. Once established, obesity is hard to reverse - for example, an estimated 90% of children with obesity at 3 y of age continue to experience overweight or obesity in adolescence [3]. Infancy is also a critical period for establishing food preferences and eating patterns that can persist into childhood and adulthood [4]. It is, therefore, crucial that policies to prevent obesity and promote healthy eating are targeted in the early years of life and that these are based on the principles of responsive feeding [5].

In England, the National Health Service (NHS) provides best practice guidelines for infant feeding [6]. In line with WHO recommendations, parents in England are advised to start weaning (introducing complementary foods) at ∼6 mo while continuing to breastfeed or give infant formula [6,7]. The NHS also advises parents not to provide snacks between meals in the first year of life and to avoid giving added sugar to infants and young children [8,9]. However, free sugars account for around 10% of total energy among children aged 1–3 y in the United Kingdom, double the recommended maximum of 5% total energy from free sugar [10]. Free sugars include all sugars added to foods by industry or consumers and sugars naturally present in honey, fruit juices, and purée [10,11].

Commercial infant foods (CIF, defined as food and drink products labeled as suitable for children ≤36 mo) are often considered a healthy choice by parents [12,13]. However, Public Health England has highlighted that the ingredients, nutrient composition, labeling, and marketing of CIF are far from ideal [12]. For example, one-third of infant meals are marketed as suitable from 4-mo [12]. Second, one-quarter of CIF snacks (defined as finger foods/snacks labeled as suitable ≤36 mo) are labeled and marketed as appropriate from 7-mo [12,14]. Third, many CIFs have a high free sugar content [15,16]. Identifying CIF with little or no free sugar can be difficult due to misleading messages and imagery on packs, including unregulated claims such as “natural” and “well-balanced” [17,18]. A survey of labeling on 3427 CIFs across 27 European countries found half included the message “no added sugar,” 35% of which contained free sugars [19].

There is concern that the information currently presented on CIF labels undermines public health guidance [12,13,20]. Proposed measures to tackle childhood obesity in the United Kingdom include ensuring that information on CIF labels is honest, transparent, and aligned with government feeding advice [21,22]. The WHO proposes using the Nutrient and Promotion Profile Model to support informed feeding decisions [14,23]. The Nutrient and Promotion Profile Model recommendations include minimum age labeling of 6-mo on all CIF, limiting the sugar content of infant meals and snacks, and adding sugar warning labels (SWLs) to products when it is not possible to include a maximum sugar content threshold, such as fruit purées [14,23]. Results of experimental studies and evidence from countries where SWLs are shown on food labels, including Chile and Mexico, show effectiveness in discouraging consumption of high-sugar foods [[24], [25], [26], [27]]. The Nutrient and Promotion Profile Model also proposes removing most nutrition, health, and marketing claims, such as “no added sugar” and “contains only natural ingredients,” which contribute to the perception that products are healthier than their nutrient composition indicates [12,23,28].

In England, families of lower socioeconomic position (SEP) tend to introduce solids at an earlier age, and children living in the most deprived areas are more than twice as likely to start primary school with obesity compared to children in the least deprived neighborhoods [2,29]. Therefore, when assessing the impact of potential policies, it is essential to consider equity of impact across socioeconomic groups.

The objective of this study was to assess the impact on parents’ infant feeding choices of *1*) increasing age guidance on CIF first foods, *2*) increasing age guidance on CIF snacks, and *3*) adding SWLs to high-sugar CIF. A secondary objective was to explore whether the impact of label changes varied by SEP. Perceptions of NHS feeding guidance were explored to provide context.

## Methods

A parallel convergent mixed-methods design was used with integration at the design level and the interpretation and reporting levels [30,31]. All study procedures were approved by the University College London ethics committee (ID: 23547/001). Data from a quantitative online survey and qualitative in-person focus groups were collected and analyzed separately. The survey quantified beliefs and feeding behaviors and included an experimental element to allow changes in infant feeding in response to different labeling scenarios to be quantified (see supplemental material for online survey and experiment questions and interview schedule). Complementary focus groups facilitated an in-depth understanding of the context and role of CIF labeling. Qualitative and quantitative results were then integrated, with each afforded equal status to provide a more comprehensive insight than would be possible from either approach individually [30,32].

### Participants

Eligibility criteria included: ≥18 y old; parent or primary caregiver of a child aged 6–23 mo; United Kingdom resident; and able to read English. Independent participant samples were recruited for the survey and interviews as both explored initial reactions to SWLs.

### Online survey and experiment

Participants were recruited and paid via a market research consultancy (Censuswide) from their online panel using previously collected information. To detect a 10% difference in the number of individuals choosing a product, with 3 groups in a between-subjects design, >392 participants per group were required. An additional 5% were recruited to account for any data discard necessary (*n* = 412); therefore, 1236 participants in total. Equal numbers of participants were recruited, with children aged 6–11 mo and 12–23 mo. Quota sampling ensured the United Kingdom’s representativeness across 6 occupation groups based on the occupational level of the household chief income earner (according to the United Kingdom Census 2020).

Randomization to experimental conditions was implemented using Censuswide’s survey platform prior to participant recruitment. The system employed a built-in random number generator to assign each respondent number to 1 of 2 conditions (to test minimum age guidance) or 1 of 3 conditions (to test SWLs), with stratification ensuring equal representation of child age groups (6–11/12–24 mo) across all conditions. Researchers (assessing outcomes) had no part in assigning conditions. Digital images of CIFs were created with parent advisors to mimic existing products.

#### Minimum age guidanc

Participants were asked to imagine their infant was 5-mo and choose 1 of 3 varieties of CIF first food (e.g., baby rice, purée) or select “none of these.” Participants were randomly assigned to view the same 3 products labeled either “4-mo+” or “6-mo+.” Participants were then asked to imagine their infant was 9-mo and choose 1 of 3 CIF snacks or select “none of these.” All 3 snacks were labeled either “6 mo+” or “12 mo+.”

#### Addition of SWL

Participants selected 1 of 3 varieties of CIF dessert or indicated “none of these.” They were randomly assigned to see the 3 desserts either without sugar labeling, with SWLs, or with SWLs and “contains natural sugar.” This was repeated with CIF snacks and the same 3 conditions.

For age and SWL experiments, participants selecting “none of these” were asked what, if anything, they would choose instead.

### Statistical analysis

IBM SPSS version 26 was used. To check the validity of the randomization process, differences in sociodemographic characteristics between label conditions were examined using χ^2^ tests (Supplemental Table 1).

Proportions and 95% confidence intervals of choosing a CIF by condition for changing age guidelines and sugar labeling were calculated. The χ^2^ tests were used to explore whether the proportion of participants choosing 1 of the 3 CIF (yes) or indicating “none of these” (no) varied according to the experimental condition. Binary logistic regression analyses were used to explore the impact of different age guidelines or sugar labeling, with the experimental condition as the independent variable (reference categories: “younger” age label or no SWL) and choosing a product (yes/no) as the dependent variable.

To assess SEP differences in responses, binary logistic regression analyses were performed with household income (or occupational level or education) as an independent continuous variable and choosing a CIF (yes/no) as the dependent variable, stratified by condition.

### Focus groups

Participants were recruited via posters and newsletters for discussion groups about “marketing and labeling of baby foods” at 2 London children’s centers. Participants received an information sheet and provided written informed consent. They completed a brief online screening survey to provide contact details and check that the inclusion criteria were met. Five focus groups were conducted with parents and accompanying children, as dictated by participant availability and available resources.

An interview schedule was developed to complement the quantitative methodology [33]. In a pile sorting activity, participants were asked to separate 20 products into foods they considered “high sugar” and “not high sugar” to prompt discussion of perceived sugar content. Black hexagonal SWLs were then stuck on high-sugar products [>30% energy from sugar (purées and desserts), >15% energy from sugar (snacks)] to understand the potential impact [23]. Lastly, 3 feeding recommendations from the NHS website were discussed to assess perceptions and provide context:

• How to start weaning – You should wait until your baby is around 6 mo old [6]
• Infants under 12 mo don’t need snacks. If you think your baby is hungry between meals, offer extra milk feeds instead [8]
• How much sugar can we eat? There’s no guideline limit for children under the age of 4, but it’s recommended they avoid sugar-sweetened drinks and food with sugar added to it [9]

**FIGURE 1:**
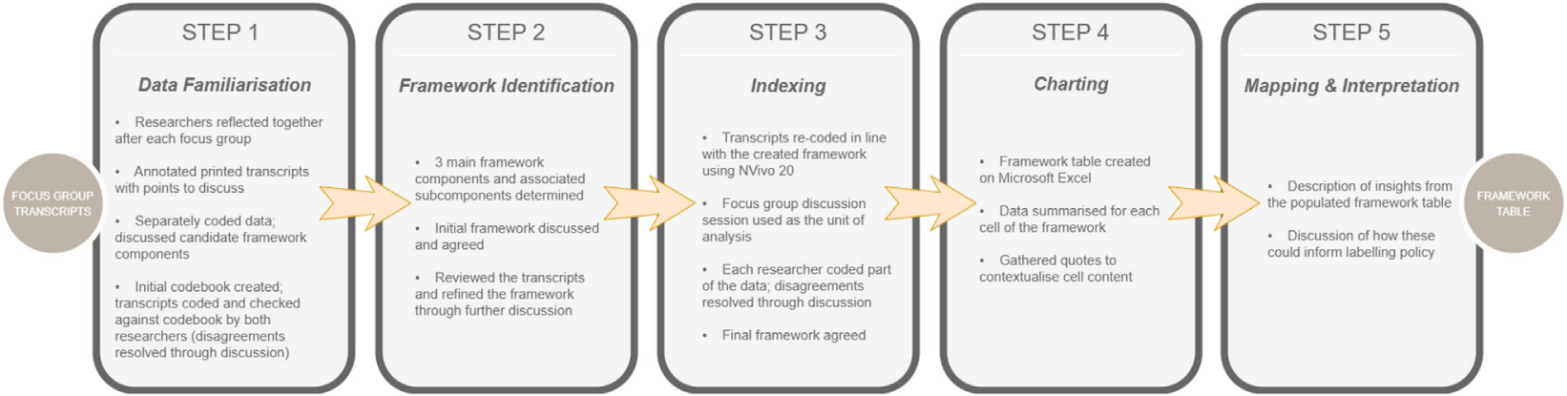
Overview of qualitative framework analysis methodology.

Focus group discussions were led by REC, with FS taking notes. REC has children, and FS does not. Both REC and FS are White female health researchers trained in qualitative methodology who were previously unknown to participants and described as “researchers.” Participants received a £25 shopping voucher. Discussions were audio recorded, transcribed, and analyzed by REC and FS using framework analysis and NVivo 20 (Figure 1) [34].

### Integration

A joint display was constructed to aid the integration of quantitative and qualitative results, as well as overall interpretation. As the purpose of mixing was complementarity, integration did not focus on identifying convergent and divergent findings but recognized both qualitative and quantitative findings as contributing to understanding by addressing similar but distinct aspects of the phenomenon [35].

## Results

### Participant characteristics

**FIGURE 2:**
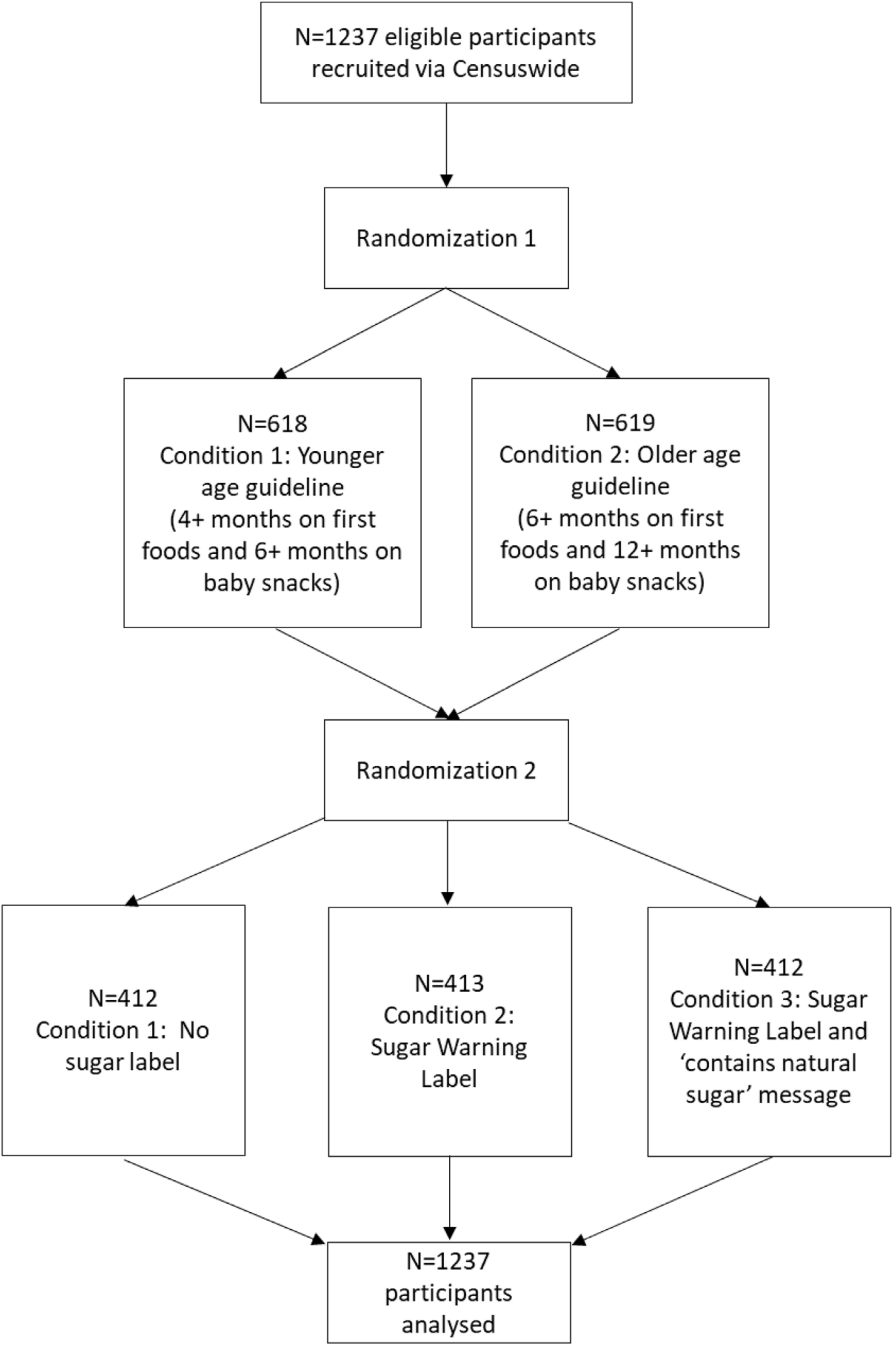
Participant flow diagram for online experiment.

Demographic characteristics are shown in Table 1. The online survey was completed by 1237 parents in October-November 2022 (Figure 2), and 22 parents took part in focus groups in November-December 2022. The focus group screening survey was completed by 30 participants, including 3 who did not reply to postscreening communications and 5 who could not attend on the day. Focus groups lasted a mean of 74 min (range 68–84). Most interviewees were mothers, and the sample was diverse in terms of socioeconomic background and ethnicity.

**TABLE 1:** Demographic characteristics of online survey and experiment participants and focus group participants.

Parental characteristics		Online survey and experiment (*n* = 1237)	Focus groups (*n* = 22)
		*n* (%)	*n* (%)
Relationship to infant	Mother	920 (74.4)	20 (90.9)
	Father	311 (25.1)	2 (9.1)
	Other main caregiver	6 (0.5)	0 (0.0)
Age (years)	18–24	146 (11.8)	0 (0.0)
	25–29	262 (21.2)	1 (4.5)
	30–34	365 (29.5)	8 (36.4)
	35–39	274 (22.2)	10 (45.5)
	40–44	124 (10.0)	3 (13.6)
	45–59	66 (5.3)	0 (0.0)
Ethnicity	White	1021 (82.5)	15 (68.2)
	Asian	93 (7.5)	2 (9.1)
	Black	56 (4.5)	3 (13.6)
	Arab	8 (0.6)	1 (4.5)
	Mixed	57 (4.6)	1 (4.5)
	Prefer not to say	2 (0.2)	0 (0.0)
Education level	Lower: none – vocational levels	250 (20.2)	3 (13.6)
	Medium: A levels- or equivalent	462 (37.3)	2 (9.0)
	Higher: bachelor – postgraduate degree	525 (42.4)	17 (77.3)
Household income^1^	Lower	236 (19.1)	2 (9.0)
	Medium	653 (52.8)	5 (22.7)
	Higher	328 (26.5)	15 (68.2)
	Prefer not to say	20 (1.6)	0 (0.0)
Household status	Married/civil partnership/living with partner	995 (80.4)	17 (77.3)
	Single parent	240 (19.4)	5 (22.7)
	Other	2 (0.2)	0 (0.0)
Number of children	1	494 (39.9)	14 (63.6)
	2	499 (40.3)	7 (31.8)
	3 or more	244 (19.7)	1 (4.5)
Infant characteristics			
Sex	Female	619 (50.0)	9 (40.9)
	Male	611 (49.4)	13 (59.1)
	Prefer not to say	7 (0.6)	0 (0.0)
Age (months)	6–11	619 (50.0)	15 (68.2)
	12–24	618 (50.0)	7 (31.8)

1 Online experiment: lower: <25k pounds per year, medium: 25–55k pounds per year, higher: >55k pounds per year. Focus groups: lower: <30k pounds per year, medium: 30–60k pounds per year, higher >60k pounds per year.

Quantitative results are shown in TABLE 2, TABLE 3, and qualitative results in Table 4. Key findings are integrated in Table 5.

**TABLE 2:** Feeding behaviors of the online survey and experiment participants.

Feeding behavior		Total sample (*n* = 1237)	Infant aged 6–11 mo (*n* = 619)	Infant aged 12–24 mo (*n* = 618)
		*n* (%)		
Introduction of complementary foods				
Believed recommended age for weaning (months)	≤3	12 (1.0)	7 (1.1)	5 (0.8)
	4–5	188 (15.2)	117 (18.9)	71 (11.5)
	6	777 (62.8)	348 (56.2)	429 (69.4)
	≥7	235 (19.0)	138 (22.3)	97 (15.7)
	Don’t know	25 (2.0)	9 (1.5)	16 (2.6)
Infant’s age when started weaning (months)^1^	≤3	38 (3.2)	28 (4.8)	10 (1.7)
	4–5	452 (38.0)	255 (43.6)	197 (32.5)
	6	512 (43.0)	213 (36.4)	299 (49.3)
	≥7	189 (15.3)	89 (15.2)	100 (16.5)
Introduction of snacks				
Believed recommended age for providing snacks between meals (months)	≤5	33 (2.7)	26 (4.2)	7 (1.1)
	6–11	858 (69.4)	462 (74.6)	396 (64.1)
	≥12	189 (15.3)	66 (10.7)	123 (19.9)
	Don’t know	157 (12.7)	65 (10.5)	92 (14.9)
Current number of snack occasions per day^1^	≤1	286 (24.0)	180 (30.8)	106 (17.5)
	2	468 (39.3)	195 (33.3)	273 (45.0)
	3	254 (21.3)	109 (18.6)	145 (23.9)
	≥4	183 (15.4)	101 (17.3)	82 (13.5)
Provide commercial infant finger foods as a snack between meals^1^^,^^2^	Yes	1042 (87.5)	485 (78.4)	557 (90.1)
Age commercial infant finger foods introduced as a snack between meals (months)^2^	≤5	308 (29.6)	189 (39.0)	119 (21.4)
	6–11	670 (64.3)	290 (59.8)	380 (68.2)
	≥12	64 (6.1)	6 (1.2)	58 (10.4)
Provide commercial infant finger foods as part of a meal^1^	Yes	603 (50.6)	332 (56.8)	271 (44.7)
Age commercial infant finger foods introduced as part of a meal^3^	≤5	230 (38.1)	151 (45.5)	79 (29.2)
	6–11	348 (28.1)	173 (52.1)	175 (64.6)
	≥12	25 (2.0)	8 (2.4)	17 (6.3)

1 Excluding those who hadn’t started weaning yet (*n* = 46).

2 Excluding those who didn’t provide commercial infant finger foods as a snack (*n* = 195).

3 Excluding those who didn’t provide commercial infant finger foods as part of a meal (*n* = 634).

**TABLE 3:** Effect of changing the age guidance and adding a sugar warning label on choosing a commercial infant food product.

Experimental label condition	Choosing a product (yes)			
	*n*	% (95% CI)	*P* value	OR (95% CI)
Age on first foods				
4 mo+ (n = 618)	544	88.0 (85.4, 90.5)	0.07	Reference
6 mo+ (n = 619)	523	84.5 (81.6, 87.2)		0.74 (0.54, 1.03)
Age on infant snacks				
6 mo+ (*n* = 618)	597	96.6 (95.1, 97.9)	<0.001	Reference
12 mo+ (n = 619)	534	86.3 (83.5, 88.9)		0.22 (0.14, 0.36)
Sugar labeling on desserts				
No sugar label (*n* = 412)	393	95.4 (93.2, 97.3)	<0.001	Reference
SWL (*n* = 413)	282	68.3 (63.7, 72.9)		0.10 (0.06, 0.17)
SWL and “contains natural sugar” (n = 412)	309	75.0 (70.6, 79.1)		0.15 (0.09, 0.24)
Sugar labeling on snacks				
No sugar label (*n* = 412)	399	96.8 (95.1, 98.3)	<0.001	Reference
SWL (*n* = 413)	342	82.8 (79.2, 86.4)		0.16 (0.09, 0.29)
SWL and “contains natural sugar” (*n* = 412)	350	85.0 (81.3, 88.3)		0.18 (0.10, 0.34)

*Note:* 95% CIs were derived with bootstrapping based on 10,000 bootstrap samples. *P* values were derived with χ^2^ tests for group differences (choosing an infant food product compared with not choosing an infant food product). OR were derived from binary logistic regression analysis, with “no” set as the reference category for the outcome.

Abbreviations: CI, confidence interval; OR, odds ratio; SWL, sugar warning label.

**TABLE 4:** Framework for analysis of focus group interviews with quotes from parents.

Themes and sub-themes	Quotes from parents
1. Age guidance for infant foods	
1.1 Perception of complementary feeding recommendations	“These are just NHS guidelines as well, so a lot of people will stick by them; others will be like, just it’s not breaking the rules, but they will just do it before the 6 months .... some people wean their baby at 3 months you know.” *R10, FG3*
1.2 Use of age guidance on infant food labels	“I started giving her little bits of food when she just turned 4 months, actually, and the first thing I chose was Ella’s Kitchen sweetcorn pouch simply because it’s 4 months and onwards.” *R3, FG1* “I waited until 6 months, whereas most of my friends said, “Well, the food says 4 months, so I’m going to do what the food company says.” *R6, FG2*
1.3 Other Factors Influencing Complementary Feeding Age	“Sometimes you feel like there’s too much information that just bombards, which is why I’ve always gone with the advice of like seeing what my family did and how they were and just based it upon my son will let me know what he needs.” *R4, FG1*
1.4 Support for changing the minimum age for first foods	“If it says 4 months, it literally gives them that green light to say, well, it says 4 months, so it’s okay. And it kind of, almost like they’ve replaced what the health visitor or the GP or the nurse has said.” *R7, FG2* “It feels like a no-brainer that the NHS guidance be reflected in everything that’s sort of publicly given, then if a doctor needs to do something different, they advise you can go against the label in this way.” R*9, FG2*
2. Age guidance on infant snacks	
2.1 Perception of recommendations for introducing snacks	“To me, that feels very specifically like they wanted to say don’t give babies snacks but felt they couldn’t, you know, because there’s all these products being sold basically.” *R9, FG2* “I think it goes hand-in-hand with the under 12 months milk, which is the main food, and the snacks and all the other foods you are giving, they said, are for fun.” *R10, FG3* “Why not? Because if they are already eating, why not have a snack?” *R19, FG4* “I took snacks to mean these packet things for me; I don’t know, or even yogurt; I would say yogurt’s a snack, then maybe it isn’t.” *R17, FG4*
2.2 Use of age guidance on infant snack labels	“For me, it was – okay, this is from 6 months, we can take it, but this is from 9 - I would rather maybe wait a little bit.” *R5, FG1"* I think, oh, is it a choking hazard? Can it melt in her mouth? Can she squeeze it? That kind of thing, then it’s okay, so I disregard a lot of the ages.” *R7, FG2* “I remember saying to my partner, “All this stuff is just marketing; they don’t need anything else until they are over 1".....by the time she was 8 months, I remember coming out of the supermarket with snacks.” *R21, FG5*
2.3 Support for changing the minimum age for infant snacks	“So, maybe, you know, just not having ages, but at the very least, I feel like the ages should reflect what medical advice says.” *R9*, *FG2*
3. Use of sugar warning labels on infant foods and snacks	
3.1 Expectations of the sugar content of infant foods	“It says ‘no added sugar’ on the box at the front; at least when you pick it up, you know already there’s no sugar in it.” *R4, FG1* “We like Ella’s Kitchen just in general; we buy it all the time because they don’t add anything except the fruits...I think it’s also because, in the UK, there are such strict rules about baby food that you automatically would think they wouldn’t put in anything harmful for children.” *R5, FG1* “It’s all put like how we would cook it and then put it in the pouch obviously minus the sugar and the salt and stuff like that.” *R16, FG4*
3.2 Understanding different types of sugar	“If I could, I’d probably split the ‘high in sugar’ into categories. I know it makes everything much more complicated, but the one with the added sugar and the one with the natural sugars...I don’t know, maybe it’s a wrong perception, but I feel like the ones that are natural sugars, the cured from fruits, for example, in my head, they’re not as bad or damaging for a child.” *R5, FG1* “Obviously, again, it’s naturally occurring sugars; it’s not too harmful.” *R22, FG5*
3.3 Reaction to SWL	“I think the ones with this label would just go out of business, basically. No one would buy them right.” *R2, FG1* “I probably would buy it as well. I could imagine that I would give it to him but maybe not a whole portion, like give him half or something just to reduce the sugar content.” *R15, FG4* “The company won’t put them on there because people won’t buy them.” *R18, FG4* “If I’d have known that high contents of sugar in, I would have never have given that to him at 2 months. Never.” R4, FG1
3.4 Support for the use of SWLs	“Yes, I think because we’re so used to seeing traffic lights in the adult range that we would get familiarity with it with the kid’s stuff, and it would naturally just be like, oh so that’s a red traffic, okay.” *R4, FG1* “I think (the sugar warning label) it’s great; I would have definitely rethought everything I brought him, and I wouldn’t have bought them (reduced sugar rusks).” *R19, FG4*
3.5 Appearance of warning label	“I want to understand what it means. So, because, for example, does it mean higher than the recommended amount of sugar they have per day or per - I don’t know, quantity per portion.” *R2, FG1* “I like the fact it’s black and white because it’s not so scary, but it is a warning if you see what I mean. So it’s sort of saying, look at me, I’m high in sugar, just so you know. So you don’t have to go; I can’t see this; I can’t read it without my glasses.” *R7, FG2*

Abbreviations: FG, focus group number; NHS, National Health Service; R, respondent number; SWL, sugar warning label; GP, General Practitioner.

**TABLE 5:** Integrated results matrix.

Concept	Key quantitative findings	Key qualitative findings	Interpretive summary
Awareness of, and adherence to, guidance to wean from ∼6 mo	63% aware of guidance. 43% weaned at 6 mo.	Interpretation of guidance varied, with many emphasizing the need for parents to decide/judge for their child.	Most parents are aware of guidance to wean at ∼6 mo and many view earlier weaning as adherent.
Potential impact of changing labels on first foods to a minimum of 6 mo+	>80% selected the first food for a 5-mo-old infant labeled 4 mo+ or 6 mo+ (88% and 85%, respectively, *P* > 0.05).	Many factors impact the age of weaning. Some chose foods labeled 4 mo+ as the first foods. Some felt pressure to wean early from parents, citing 4 mo+ labels as evidence of appropriateness. Parents felt consistent messaging would be helpful.	May not delay weaning from 5 mo to 6 mo for many, but it could provide clarity and also reduce the pressure some feel to wean before 6 mo.
Awareness of and adherence to guidance to introduce snacks only from 12-mo	15% aware of guidance. 88% gave CIF snacks before 12 mo.	Most parents were unaware of guidance and, when shown, felt it wasn’t clearly written. Most used CIF snacks regularly before 12 mo, particularly on the go.	Use of CIF snacks <12 mo is the norm. Most parents haven’t seen guidance and think it’s unclear.
Potential impact of changing labels on snacks to a minimum of 12 mo+	Fewer participants selected a snack labeled 12 mo+ (87%) than 6 mo+ (97%) for an infant aged 9 mo.	Snacks are considered appropriate and fulfill a valued role (e.g., entertaining infants). Some always strictly follow age guidance. Some would disregard age labels if products were marketed/labeled as appropriate (e.g., “melty”).	May reduce snack use <12 mo, but the impact is likely to be limited without increased awareness of guidance and other labeling and marketing changes.
Awareness of the high sugar content of some infant food products	The majority believed infant products had no added sugar (70%) and were low in sugar (63%).	Content of CIF was thought to be regulated and, therefore, assumed products were appropriate. Many shocked products were high in sugar.	Low awareness that infant products often have a high sugar content.
Potential impact of adding SWLs	Desserts with a SWL label chosen by 27% fewer parents but concurrently displaying “contains natural sugar” reduced the impact of SWL.	Support for SWLs or similar labels and belief this would reduce feeding frequency or portions. Some wanted additional information about “added” or “natural” sugar, viewing the former as less healthy/more problematic.	Adding SWLs to high-sugar CIF has the potential to reduce infants' sugar intakes, but additional insight is needed around terminology such as “natural sugar.”

Abbreviations: CIF, commercial infant food; SWL, sugar warning label.

### Minimum age guidance of first foods

Most survey participants were aware of official guidance to wean from ∼6-mo (62.8%), but only 43.0% started weaning at 6-mo. Although some focus group interviewees described following the guidance by weaning at 6-mo, others started earlier as they didn’t perceive the guidance as a precise instruction, instead emphasizing the importance of parents’ assessing their baby’s individual readiness (theme 1.1). A similar proportion of survey participants indicated they would buy a first food for a 5-mo-old infant labeled “4-mo+” or “6-mo+” (88.0% compared with 84.5%, *P* = 0.07). More than 80% of those not choosing a product indicated they would give a 5-mo-old infant breast- or formula milk instead. Some focus group interviewees described weaning before 6-mo with products labeled “4-mo+” and others described feeling pressure to wean before 6-mo from fellow parents who cited “4-mo+” labels as evidence that this was appropriate (theme 1.2). Interviewees described a range of factors influencing the timing of weaning, including mothers, mothers-in-law, friends, and information from infant food brands, especially those with nutritionists, as these were perceived as particularly trustworthy (theme 1.3). Interviewees described “4-mo+” labels as confusing, misleading, or deceptive (theme 1.4). Written age guidance on labels was seen by some as carrying more weight than verbal advice from healthcare professionals. Parents wanted consistent advice and strongly supported minimum age guidance of “6-mo+” on CIF first foods.

### Minimum age guidance on CIF snacks

Only 13.5% of survey respondents correctly identified 12-mo as the recommended age for introducing snacks, with 72.1% believing snacks should be introduced earlier. Likewise, focus group interviewees were unaware of age guidance, and even when presented with the NHS snack guidance, uncertainty remained (theme 2.1). Several interpreted NHS advice as meaning milk should be the main source of nutrition for the first year and that food was less important. Some interpreted “snacks” as anything additional to infants' 3 daily meals, including fruit, whereas others felt the advice was specifically about unhealthy snacks, such as chips and biscuits. Interviewees agreed that the advice was unclear and needed to be more specific.

Survey results showed most infants aged 6–11-mo currently consumed CIF snacks (78.4%), and many had started before 6-mo (39.0%). In line with this, interviewees gave CIF snacks before 12-mo as they thought age guidance on labels meant this was recommended (theme 2.2). Some also felt younger age guidance indicated lower sugar content, as younger infants were not allowed sugar. The wide availability of CIF snacks contributed to perceptions that snacks were recommended before 12 mo. One mother recalled that she had intended not to give snacks until 12-mo, then found herself leaving the supermarket with snacks for her 8-mo-old infant. If the snacks were labeled “12-mo+,” she said she would not have bought them.

When presented with CIF snacks labeled “12-mo+,” fewer survey respondents selected one to give a 9-mo-old infant, compared to those presented with identical products labeled “6-mo+” (86.3% compared with 96.3%, *P* < 0.001). Participants indicating they would not buy CIF snacks labeled “12-mo+” reported that instead, they would give fresh fruit (49.4%), breast- or formula milk (29.4%), or another snack (11.8%). Some focus group interviewees said they might ignore “12-mo+” guidance if snacks were “melty” and a good shape for self-feeding by younger infants as they knew from experience that these were suitable for younger infants (theme 2.3). Interviewees wanted age guidance on CIF snacks to be consistent with NHS snack guidance.

### Addition of a SWL

**FIGURE 3:**
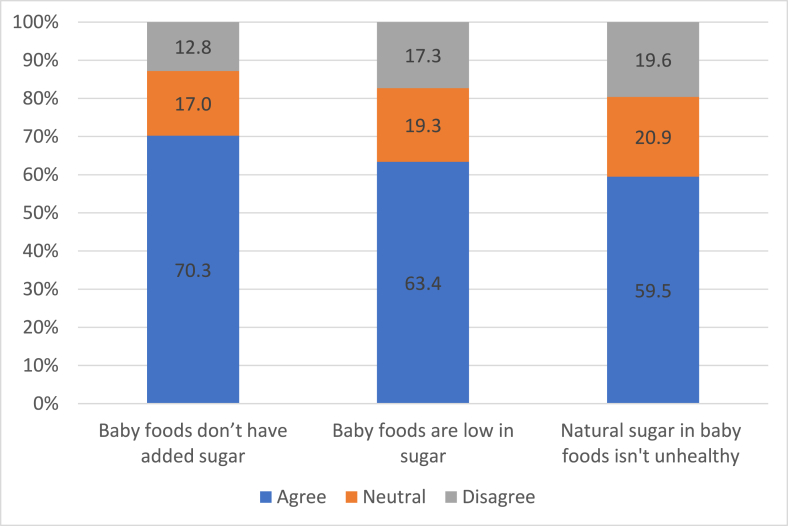
Beliefs about sugar in infant food from parents participating in the online survey and experiment (*n* = 1237).

Most survey participants believed CIF do not contain added sugar (70.3%) and are low in sugar (59.5%) (Figure 3). Focus group interviewees expanded on this, describing CIF as being strictly regulated and therefore not containing unhealthy concentrations of sugar, with brand trust and marketing confirming for them that products were appropriate and healthy for infants (theme 3.1). This was highlighted in the pile sorting activity, where cookies and chocolate marketed for older children were immediately dismissed as high sugar and unsuitable for infants. No CIF was dismissed in this way. Most survey participants believed natural sugar in CIF wasn’t bad for infants (59.5%), and interviewees described “natural sugar” or “fruit sugar” as being different and not as bad as “actual sugar” or “refined sugar” (theme 3.2). Interviewees were often drawn to CIF labeled “no added sugar” or “reduced sugar,” but some expressed confusion over the meaning of these terms.

CIF desserts displaying a SWL were chosen by 27.1% fewer participants than identical desserts without a SWL (68.3% compared with 95.4%) (Table 3). The impact of SWLs was reduced when “contains natural sugar” was also displayed, with 75.0% of parents then choosing a dessert. Participants not choosing a dessert with a SWL (31.7%) often said they would give fresh fruit (75.6%), yogurt (8.4%), or dried fruits (4.6%) instead. Similarly, infant snacks displaying a SWL were chosen by 14.0% fewer parents than identical snacks without a SWL (82.8% compared with 96.8%), and 85.0% chose a snack if the message “contains natural sugar” was also displayed. Participants not choosing CIF snacks with an SWL (17.2%) said they would give fruit or vegetables (46.8%), savory CIF snacks (31.0%), and savory snacks marketed for children (11.3%) instead. Focus group interviewees were generally shocked when SWL were added to products (theme 3.3). Some expressed disbelief, and 1 mother felt deceived and regretted giving rusks assigned a SWL to her son at 2-mo; she believed these didn’t contain sugar because they were labeled “reduced sugar.” Parents felt SWLs would stop them from buying some products, reduce the frequency or portion size they would give, or at least make them “stop and think.”

In focus groups, there was strong and unanimous support for adding SWLs to CIF (theme 3.4). Interviewees emphasized a need for these to be front-of-pack and large enough to be obvious when quickly scanning shelves. Although the SWL shown was considered appropriate by many parents, alternative formats were also suggested, such as using a different color (not black) or traffic light labeling, which they felt more familiar with (theme 3.5). Some parents wanted the SWL to distinguish between added sugar, which they wanted to avoid, and natural sugar, which they considered healthier. Several said SWLs would prompt them to inspect ingredient lists to see if “sugar” was listed or if the product contained “just fruit or juice,” which would indicate to them that it wasn’t really unhealthy.

### SEP

In the survey, no associations were observed between household income level and the odds of choosing a product with a particular age or sugar label (Supplemental Tables 2 and 3). Repeating analysis with occupation or education level produced similar results. Qualitative results were not analyzed using participants’ SEP.

## Discussion

The results of this mixed-methods study suggest that increasing age guidance and applying SWLs to CIF could shift feeding behaviors toward closer alignment with NHS guidance. Integrated findings demonstrate an overall baby aisle halo” effect, with CIF believed to be strictly regulated and therefore healthy, which conflicts with analysis showing many products have a high sugar content [12,16,36].

In line with previous findings from Public Health England, ∼2 in 5 participants started weaning before the recommended age of 6-mo [37]. Results from the online experiment may initially appear incongruous with focus group results as the experiment did not demonstrate differences in the proportion of participants choosing products labeled “6-mo+” or “4-mo+” (Table 3), whereas parents in focus groups viewed “4-mo+” labels as problematic and supported “6-mo+” labels being the minimum permitted. Survey participants were asked to imagine their infant was 5-mo, which many may have viewed as being “∼6-mo.” The qualitative methodology facilitated a more nuanced understanding of the potential impact of regulating age guidance and aligns with findings from qualitative research in Australia, in which parents felt a minimum of “6-mo+” labels would support them to feed in line with best practice guidance [38]. Parents in the current study and others point to a range of factors influencing the timing of introducing solids [37,38]. Parents awareness of advice to wean around 6-mo but emphasis on assessing infants’ individual readiness may stem from family and friends but also CIF brand websites, which promote the idea that readiness is associated with advanced development, thereby encouraging early introduction of solids [7,39,40].

More than a third of participants provided 3 or more snacks per day, which is a concern as increased snack frequency is associated with higher energy intake in toddlers [41]**.** Providing infants (<12 mo) with multiple daily snacks was the norm, and results suggest making “12-mo+” the minimum permissible age guidance could have a positive impact. CIF snacks are often labeled as “finger foods” and could theoretically be consumed at mealtimes and display “6-mo+” labels, but most CIF finger foods resemble regular snack products (e.g., cookies and savory puffs). In focus groups, parents referred to CIF snack/finger foods as “snacks” and “on the go” foods and 87.5% of survey participants reported giving them between meals (Table 2). Parents found inconsistent messaging particularly difficult to navigate. Although the NHS advises introducing “snacks” ≥12-mo, they recommend “finger foods” from around 6-mo when solids are introduced, for example, “*soft cooked vegetables*” and “*hardboiled eggs*,” which may add to caregivers’ confusion [6]. Results suggest that NHS advice to feed 2 healthy snacks per day from 12-mo, such as fruit or vegetables, should be clearly and consistently communicated by healthcare professionals, on infant food brand websites, and in other communications [6]. Previous qualitative research has found parents feel products labeled as “melty” or “encourage self-feeding” are suitable for very young infants, and in the current study, parents said they might ignore age guidance if they viewed products as appropriate for younger infants, which supports WHO calls to prohibit the use of these promotional messages [13,23]. Results highlight the need to clearly define “snacks” both in research and in communications regarding infant feeding. Clear communication is also needed regarding the importance of early and repeated exposure to healthy, unprocessed foods, which is an important element of the responsive feeding approach, to encourage a preference for these foods [5].

The smaller proportion of participants choosing high-sugar products when SWLs were displayed aligns with findings that fewer parents would give high-sugar beverages to children aged <5 y if SWLs were displayed [23,27]. There is, however, a potential risk of shifting choices toward higher-sugar alternatives. Although fresh fruit was the most popular alternative dessert reported by parents, yogurts were the next choice. Flavored yogurts are often high in free sugars and marketed as infant foods due to their size and the use of characters and graphics but are not specifically labeled as suitable ≤36-mo, which means if SWL were added only to CIF, these yogurts would not display them. The WHO suggests such products should state a minimum age of 36-mo/3-y on pack [23].

The dampening effect of simultaneously displaying “contains natural sugar” with a SWL is in line with findings from experimental studies with adult consumers, suggesting SWLs may mitigate but not eliminate the influence of nutrient-related claims, thereby supporting calls for such “health halo” messages to be regulated [26,29,42,43]. Parents’ beliefs about “natural sugar” not being problematic require further investigation as it is at odds with WHO and the United Kingdom guidance, which recommends limiting free sugars, whether these are extracted from sugar cane or fruit [7,10]. It is currently unclear how best to address misperceptions about sugar terminology on labels [44]. Although some parents expressed a preference for traffic light labeling, these are technically problematic for CIF as nutrient recommendations vary with children’s age. Furthermore, meta-analyses show that warning labels are more effective than traffic light labels in discouraging unhealthy purchasing behavior [24,26].

Results of analyses by SEP support evidence suggesting front-of-pack labels are an equitable public health intervention [45]. In focus groups, researchers noted that interviewees with higher occupational status sometimes described active information seeking, which resulted in them being more skeptical about information provided by CIF companies. In addition to labeling changes, the WHO proposes setting limits for the sugar content of products (i.e., reformulation), which could be more effective than label changes in reducing inequalities in diet and weight as the need for individual agency in selecting appropriate products is reduced [14,46].

Overall, findings support specific label changes as part of a comprehensive policy toolkit to provide parents with agency to improve the diets of their infants and young children [14]. In Chile, the introduction of SWLs and marketing restrictions was associated with a 26.7% decline in sugar purchased from products with a SWL and an overall reduction in sugar purchases of 10.2%, according to a before and after study using household purchasing data [47]. However, there is evidence that SWLs face resistance and pushback from the industry, and the WHO highlights a wide range of evidence that industry opposition is a major barrier to implementing new nutrient labeling policies [26,48]. Nonetheless, the United Kingdom government has made a commitment to making CIF labels clearer, as well as highlighting the United Kingdom’s decision to leave the European Union, which provides greater flexibility in doing so [49]. Findings also highlight a need for clearer and more consistent public health advice around providing snacks to infants and young children.

Integrating focus group interviews with quantitative results from a large representative sample of caregivers provided an in-depth understanding of how parents use CIF labels and might react to mandatory label changes. However, the findings have some limitations, and the ecological validity of the online experiment must be considered. For example, mock product images were used to reduce bias related to brand perception, and although parent advisors contributed to label designs, this may have affected choices. Also, participants were unable to view back-of-pack information, which could influence choices in a real-life context. In retrospect, when assessing the impact of “4-mo+” compared with “6-mo+” labels, it may have been preferable to ask parents to imagine their infant was 3- or 4-mo rather than 5-mo. Also, it is unclear how asking parents to imagine their child was a particular age may have impacted results. As parents already fed CIF snacks (e.g., wafers) to their infants, they felt comfortable that these were appropriate for younger infants; therefore, the impact of increasing age guidance may have been underestimated. A further limitation was the exclusion of people who were not able to read English, which may limit the external validity of the findings. It may have been preferable to include a single population sample in both the survey and interviews, but it was not practical to bring together survey participants from across the country, and we aimed to assess initial reactions to SWLs in both.

In conclusion, results demonstrate that ensuring minimum age guidance of 6-mo on CIF first foods and 12-mo on CIF snacks could shift feeding behaviors toward closer alignment with government feeding advice. Findings also suggest adding SWLs to front-of-packs and removing “contains natural sugar” could support a reduction in sugar intake. Importantly, the impact of these label changes appears socioeconomically equitable, and they are universally supported by parents. Consistent messaging from companies and health professionals, including online and via social media, is also required to support parents in making healthy food choices on behalf of their infants.

## Author contributions

The authors’ responsibilities were as follows– REC, CHL: conceptualized the study; REC, FS, CHL: designed the study; REC, FS: coordinated the online survey and experiment and conducted focus groups; ID conducted statistical analysis in consultation with REC and CL; REC: wrote the initial draft of the article with input from all authors; and all authors: read and approved the final manuscript.

## Data availability

The data sets used and/or analyzed during the current study are available from the corresponding author upon reasonable request.

## Funding

This study was funded by the National Institute for Health and Care Research (NIHR) (PR- PRU- 0916- 21001). The views expressed are those of the author(s) and not necessarily those of the NIHR or the Department of Health and Social Care. The funder had no role in data collection, data analysis, interpretation, or manuscript preparation.

## Conflict of interest

REC, ID and FS report financial support was provided by National Institute of Health and Social Care. Other authors declare that they have no known competing financial interests or personal relationships that could have appeared to influence the work reported in this article.
