# Supplementary material for: Estimating the Impact on Parents’ Infant Feeding Choices of Increasing Age Guidance and Adding Sugar Warning Labels to Commercial Infant Foods: A Mixed-Methods Study
Source: Curr Dev Nutr. 2025 Apr 15;9(5):107443. doi: 10.1016/j.cdnut.2025.107443 (PMC12147840; doi:10.1016/j.cdnut.2025.107443)
Supplement: multimedia component 1 [file mmc1.docx]

**Supplementary materials**

**Estimating the impact on parents’ infant feeding choices of increasing age guidance and adding sugar warning labels to commercial infant foods: a mixed methods study**

Rana E. Conway

**Table S1. Characteristics of the online survey sample and by experiment conditions.**

|  |  |  | **Two conditions** | | | **Three conditions** | | | |
| --- | --- | --- | --- | --- | --- | --- | --- | --- | --- |
| **Parental characteristics** |  | **Total sample (n=1237)** | **Condition 1 (n=618)** | **Condition 2 (n=619)** | **p-value*** | **Condition 1 (n=412)** | **Condition 2**  **(n=413)** | **Condition 3**  **(n=412)** | **p-value*** |
| Relationship to baby/toddler | Mother | 920 (74.4%) | 462 (74.8%) | 458 (74.0%) | 0.70 | 301 (73.1%) | 312 (75.5%) | 307 (74.5%) | 0.78 |
|  | Father | 311 (25.1%) | 154 (24.9%) | 157 (25.4%) |  | 109 (26.5%) | 98 (23.7%) | 104 (25.2%) |  |
|  | Other main caregiver | 6 (0.5%) | 2 (0.3%) | 4 (0.6%) |  | 2 (0.5%) | 3 (0.7%) | 1 (0.2%) |  |
| Ethnicity | White | 1021 (82.5%) | 517 (83.7%) | 504 (81.4%) | 0.38 | 338 (82.0%) | 343 (83.1%) | 340 (82.5%) | 0.50 |
|  | Asian | 93 (7.5%) | 43 (7.0%) | 50 (8.1%) |  | 28 (6.8%) | 36 (8.7%) | 29 (7.0%) |  |
|  | Black | 56 (4.5%) | 31(5.0%) | 25 (4.0%) |  | 20 (4.9%) | 13 (3.1%) | 23 (5.6%) |  |
|  | Arab | 8 (0.6%) | 2 (0.3%) | 6 (1.0%) |  | 5 (1.2%) | 2 (0.5%) | 1 (0.2%) |  |
|  | Mixed | 57 (4.6%) | 25 (4.0%) | 32 (5.2%) |  | 19 (4.6%) | 19 (4.6%) | 19 (4.6%) |  |
|  | Prefer not to say | 2 (0.2%) | 0 (0.0%) | 2 (0.3%) | 0.49 | 2 (0.5%) | 0 (0.0%) | 0 (0.0%) |  |
| Education level | None – vocational levels | 250 (20.2%) | 121 (19.6%) | 129 (20.8%) | 0.74 | 80 (19.4%) | 85 (20.6%) | 85 (20.6%) | 0.75 |
|  | A levels- HNC, HND | 462 (37.3%) | 237 (38.3%) | 225 (36.3%) |  | 154 (37.4%) | 146 (35.2%) | 162 (39.3%) |  |
|  | Bachelor – postgraduate degree | 525 (42.4%) | 260 (42.1%) | 265 (42.8) |  | 178 (43.2%) | 182 (44.1%) | 165 (40.0%) |  |
| Household income | <25k | 236 (19.1%) | 121 (19.6%) | 115 (18.6%) | 0.84 | 86 (20.9%) | 75 (18.2%) | 75 (18.2%) | 0.68 |
|  | 25k -55k | 653 (52.8%) | 327 (52.9%) | 326 (52.7%) |  | 206 (50.0%) | 226 (54.7%) | 221 (53.6%) |  |
|  | >55k | 328 (26.5%) | 160 (25.9%) | 168 (27.1%) |  | 113 (27.4%) | 105 (25.4%) | 110 (26.7%) |  |
|  | Prefer not to say | 20 (1.6%) | 10 (1.6%) | 10 (1.6%) |  | 7 (1.7%) | 7 (1.7%) | 6 (1.5%) |  |
| Household composition | Two parents | 995 (80.4%) | 501 (81.1%) | 494 (79.8%) | 0.85 | 327 (79.4%) | 334 (80.9%) | 334 (81.1%) | 0.33 |
|  | Single parent | 240 (19.4%) | 116 (18.8%) | 124 (20.0%) |  | 85 (20.6%) | 79 (19.1%) | 76 (18.4%) |  |
|  | Other | 2 (0.2%) | 1 (0.2%) | 1 (0.2%) |  | 0 (0.0%) | 0 (0.0%) | 2 (0.5%) |  |
| Number of children | 1 | 494 (39.9%) | 238 (38.5%) | 256 (41.4%) | 0.54 | 158 (38.3%) | 174 (42.1%) | 162 (39.3%) | 0.85 |
|  | 2 | 499 (40.3%) | 258 (41.7%) | 241 (38.9%) |  | 171 (41.5%) | 161 (39.0%) | 167 (40.5%) |  |
|  | 3 or more | 244 (19.7%) | 122 (19.7%) | 122 (19.7%) |  | 83 (20.1%) | 78 (18.9%) | 83 (20.1%) |  |
| **Infant characteristics** |  |  |  |  |  |  |  |  |  |
| Gender | Female | 619 (50.0%) | 323 (52.3%) | 296 (47.8%) | 0.29 | 213 (51.7%) | 206 (49.9%) | 200 (48.5%) | 0.72 |
|  | Male | 611 (49.4%) | 292 (47.2%) | 319 (51.5%) |  | 196 (47.6%) | 204 (49.4%) | 211 (51.2%) |  |
|  | Prefer not to say | 7 (0.6%) | 3 (0.5%) | 4 (0.6%) |  | 3 (0.7%) | 3 (0.7%) | 1 (0.2%) |  |
| Age | 6-11 months | 619 (50.0%) | 309 (50.0%) | 310 (50.1%) | 0.98 | 206 (50.0%) | 207 (50.1%) | 206 (50.0%) | 1.00 |
|  | 12-24 months | 618 (50.0%) | 309 (50.0%) | 309 (49.9%) |  | 206 (50.0%) | 206 (49.9%) | 206 (50.0%) |  |

* p-values were obtained with Chi Square tests.

**Table S2. Feeding behaviours of the online survey participants by household income level (n=1217).**

|  |  | **Household income^1^** | | |  |
| --- | --- | --- | --- | --- | --- |
|  |  | **Lower**  **(n=236)** | **Medium**  **(n=653)** | **Higher**  **(n=328)** | **p-value^2^** |
| **Feeding behaviour** |  | **n (%)** | | |  |
| *Introduction of complementary feeding* |  |  |  |  |  |
| Believed recommended age for introduction of complementary feeding | 3 months or younger | 2 (0.8) | 6 (0.9) | 4 (1.2) | 0.11 |
|  | 4 -5 months | 37 (15.7) | 111 (17.0) | 39 (11.9) |  |
|  | 6 months | 155 (65.7) | 410 (62.8) | 196 (59.8) |  |
|  | 7 months or older | 38 (16.1) | 113 (17.3) | 82 (25.0) |  |
|  | I don’t know | 4 (1.7) | 13 (2.0) | 7 (2.1) |  |
| Age complementary foods were introduced^3^ | 3 months or younger | 6 (2.7) | 24 (3.8) | 8 (2.6) | 0.09 |
|  | 4-5 months | 93 (41.7) | 250 (39.3) | 101 (32.3) |  |
|  | 6 months | 84 (37.7) | 273 (42.9) | 146 (46.6) |  |
|  | 7 months or older | 40 (17.9) | 89 (14.0) | 58 (18.5) |  |
| *Introduction of snacks* |  |  |  |  |  |
| Believed recommended age for providing snacks between meals | 5 months or younger | 8 (3.4) | 13 (2.0) | 12 (3.7) | 0.002 |
|  | 6 months -11 months | 173 (73.3) | 462 (70.8) | 213 (64.9) |  |
|  | 12 months or older | 24 (10.2) | 90 (13.8) | 71 (21.6) |  |
|  | I don’t know | 31 (13.1) | 88 (13.5) | 32 (9.8) |  |
| Current number of snack occasions per day^3^ | 1 occasion or less | 57 (25.6) | 152 (23.9) | 72 (23.0) | 0.86 |
|  | 2 occasions | 88 (39.5) | 241 (37.9) | 131 (41.9) |  |
|  | 3 occasions | 42 (18.8) | 143 (22.5) | 63 (20.1) |  |
|  | 4 occasions or more | 36 (16.1) | 100 (15.7) | 47 (15.0) |  |
| Provide commercial baby finger foods as a snack between meals^3^ | Yes | 202 (90.6) | 559 (87.9) | 264 (84.3) | 0.09 |
| Age commercial baby finger foods as a snack between meals were introduced^4^ | 5 months or younger | 54 (30.7) | 170 (30.4) | 75 (28.4) | 0.97 |
|  | 6-11 months | 129 (63.9) | 355 (63.5) | 172 (65.2) |  |
|  | 12 months or older | 11 (5.4) | 34 (6.1) | 17 (6.4) |  |
| Provide commercial baby finger foods as part of a meal^3^ | Yes | 97 (43.5) | 326 (51.3) | 175 (55.9) | 0.018 |
| Age commercial baby finger foods as part of a meal were introduced^5^ | 5 months or younger | 38 (39.2) | 120 (36.8) | 72 (41.1) | 0.37 |
|  | 6-11 months | 55 (56.7) | 197 (60.4) | 93 (53.1) |  |
|  | 12 months or older | 4 (4.1) | 9 (2.8) | 10 (5.7) |  |

^1^Annual household income. Low: £25000 or less. Medium: £25001-£55000. High: £55001 or more. N=20 participants did not provide information on household income (‘prefer not to say’).
^2^Differences between groups were tested with Chi-Square tests.
^3^Excluding those who hadn’t started weaning yet (n=45).
^4^Excluding those who didn’t provide commercial baby finger foods as a snack (n=192).
^5^Excluding those who didn’t provide commercial baby finger foods as part of a meal (n=619).

**Table S3. The effect of *household income level* on choosing an infant food product, within online experiment condition groups.**

|  | **Choosing an infant food product (yes)** | |
| --- | --- | --- |
| **Online experiment condition groups** | **OR (95% CI)** | **p-value** |
| *Age on first foods* |  |  |
| 4 months and older (n=608) | 1.05 (0.91,1.20) | 0.52 |
| 6 months and older (n=609) | 0.99 (0.89, 1.11) | 0.88 |
| *Age on baby snacks* |  |  |
| 6 months and older (n=608) | 1.17 (0.91, 1.50) | 0.22 |
| 12 months and older (n=609) | 1.08 (0.95, 1.21) | 0.23 |
| *Sugar labelling on desserts* |  |  |
| No sugar label (n=405) | 0.98 (0.77, 1.23) | 0.83 |
| SWL (n=406) | 0.94 (0.84, 1.05) | 0.25 |
| SWL & ‘contains natural sugar’ (n=406) | 0.99 (0.88, 1.11) | 0.85 |
| *Sugar labelling on snacks* |  |  |
| No sugar label label (n=405) | 1.02 (0.77, 1.35) | 0.90 |
| SWL (n=406) | 0.91 (0.79, 1.05) | 0.20 |
| SWL & ‘contains natural sugar’ (n=406) | 1.07 (0.93, 1.27) | 0.35 |

*Note:* Odds Ratios were derived from binary logistic regression analysis, with ‘no’ set as reference category for the outcome. Household income level consisted of 8 categories and was included as a continues variable ranging from low to high. N=20 participants had missing data on household income (answered ‘prefer not to say’). SWL: Sugar Warning Label

**Online survey and experiment questions**

Qi. How old are you?

*Drop down selection of 16-100+*

*END if aged 16-17*

Qa. Which of the following family members do you have? (Tick all that apply)

Parent(s)

Grandparent(s)

Aunt(s)

Uncle(s)

Brother(s)

Sister(s)

Cousin(s)

Child(ren)

None of the above *exclusive*

[END if ‘Child(ren)’ is not selected]

Qb. How old is / are your child(ren)? (Tick all that apply)

Less than 6 months old

6-11 months old

12-23 months old

2-4 years old

5-9 years old [Hide if respondent is under 20]

10-17 years old [Hide if respondent is under 25]

18-21 years old [Hide if respondent is under 33]

22-25 years old [Hide if respondent is under 37]

26+ [Hide if respondent is under 41]

[END if ‘6-11 months old’ or ‘12-23 months old’ is not selected]

Qc. What best describes the occupation of the Chief Income Earner in your household? (i.e. person with the highest income)

If the Chief Income Earner is now retired, please consider their occupation before retirement

Higher managerial, administrative or professional

Intermediate managerial, administrative or professional

Supervisory or clerical, junior managerial, administrative or professional

Skilled manual workers

Semi-skilled and unskilled manual workers

Casual or lowest grade workers, pensioners, and others who depend on the welfare state for their income

Each option corresponds to the following

A

B

C1

C2

D

E

Qii. What is your gender?

Male

Female

Non-binary

Not listed (please specify)

Qiii. What is your ethnic group or background?

White - English / Welsh / Scottish / Northern Irish / British

White - Irish

White - Gypsy or Irish Traveller

White - Any other White background

Mixed descent - White and Black African

Mixed descent - White and Black Caribbean

Mixed descent - White and Asian

Mixed decent - Any other mixed

Asian - Indian

Asian - Pakistani

Asian - Bangladeshi

Asian - Chinese

Any other Asian ethnic group

Black - African

Black - Caribbean

Any other Black / African / Caribbean ethnic group

Arab

Not listed (please specify)

Prefer not to say

Qiv. How would you describe your household?

Single parent

Two parent household

Not listed (please specify)

Qv. What is the highest level of education you have achieved?

GCSE / O-levels

A – Levels

Bachelor degree

DipHE

Diploma

Foundation degree

HNC or HND

International Baccalaureate Diploma

Postgraduate qualifications

Advanced Extension Awards

BTECs, OCR Nationals / Vocational qualifications

None of the above

Qvi. Which of the following best reflects your annual household income?

£15,000 or less

£15,001 – £25,000

£25,001 – £35,000

£35,001 – £45,000

£45,001 – £55,000

£55,001 – £65,000

£65,001 – £75,000

Over £75,000

I do not wish to divulge this information

Qvii. How many children do you have?

1

2

3

4 or more

*Cannot select less than the number of options selected at Qb i.e. if I select two options at Qb I cannot select 1 here.

Caveat:

Some of the following questions concern your child aged 6-23 months.

If you have more than one child aged 6-23 months, please complete the rest of this survey thinking about your youngest child in this age group. [Hide if respondents only select '1' at Qvii]

Qviii. How old is your baby/toddler (in months)?

6-7 [Hide if ‘6-11 months old’ is not selected at Qb]

8-9 [Hide if ‘6-11 months old’ is not selected at Qb]

10-11 [Hide if ‘6-11 months old’ is not selected at Qb]

12-14 [Hide if ‘12-23 months old’ is not selected at Qb]

15-16 [Hide if ‘12-23 months old’ is not selected at Qb]

17-18 [Hide if ‘12-23 months old’ is not selected at Qb]

19-21 [Hide if ‘12-23 months old’ is not selected at Qb]

22-23 [Hide if ‘12-23 months old’ is not selected at Qb]

Qix. What is your baby/ toddler’s gender?

Male

Female

Prefer not to say

Qx. What best describes your relationship to the baby/toddler?

Mum

Dad

Other main carer – please specify

Qxi. What dietary requirements, if any, does your baby/toddler have? (Tick all that apply)

None *exclusive*

Vegetarian

Vegan

Halal

Gluten-free

Nut-free

Dairy-free

Other (please specify)

[***Quotas: 1236 respondents in total:***

618 respondents need to be parents/caregivers for 6-11mo

618 respondents need to be parents/caregivers for 12-23mo

50 respondents in Higher managerial, administrative and professional SEG

284 respondnets in Intermediate managerial, administrative and professional SEG

346 respondnets in Supervisory, clerical and junior managerial, administrative and professional SEG

247 respondnets in Skilled manual workers SEG

185 respondnets in Semi-skilled and unskilled manual workers SEG

124 respondnets in State pensioners, casual and lowest grade workers, unemployed with state benefits only SEG

At Q1-Q5 and Q8-Q10.2 - quotas needed for parents to see each condition:

Condition 1: 309 respondents who are parents/caregivers for 6-11mo

Condition 2: 309 respondents who are parents/caregivers for 6-11mo

Condition 1: 309 respondents who are parents/caregivers for 12-23mo

Condition 2: 309 respondents who are parents/caregivers for 12-23mo

At Q1-Q5 and Q8-Q10.2 - quotas needed for SEG to see each condition:

Condition 1: 25 respondents who are in Higher managerial, administrative and professional SEG

Condition 2: 25 respondents who are in Higher managerial, administrative and professional SEG

Condition 1: 142 respondents who are in Intermediate managerial, administrative and professional SEG

Condition 2: 142 respondents who are in Intermediate managerial, administrative and professional SEG

Condition 1: 173 respondents who are in Supervisory, clerical and junior managerial, administrative and professional SEG

Condition 2: 173 respondents who are in Supervisory, clerical and junior managerial, administrative and professional SEG

Condition 1: 123 respondents who are in Skilled manual workers SEG

Condition 2: 124 respondents who are in Skilled manual workers SEG

Condition 1: 92 respondents who are in Semi-skilled and unskilled manual workers SEG

Condition 2: 93 respondents who are in Semi-skilled and unskilled manual workers SEG

Condition 1: 62 respondents who are in State pensioners, casual and lowest grade workers, unemployed with state benefits only SEG

Condition 2: 62 respondents who are in State pensioners, casual and lowest grade workers, unemployed with state benefits only SEG

At Q6-Q7 - quotas needed for parents to see each condition:

Condition 1: 206 respondents who are parents/caregivers for 6-11mo

Condition 2: 206 respondents who are parents/caregivers for 6-11mo

Condition 3: 206 respondents who are parents/caregivers for 6-11mo

Condition 1: 206 respondents who are parents/caregivers for 12-23mo

Condition 2: 206 respondents who are parents/caregivers for 12-23mo

Condition 3: 206 respondents who are parents/caregivers for 12-23mo

At Q6-Q7 - quotas needed for SEG to see each condition:

Condition 1: 16 respondents who are in Higher managerial, administrative and professional SEG

Condition 2: 17 respondents who are in Higher managerial, administrative and professional SEG

Condition 3: 17 respondents who are in Higher managerial, administrative and professional SEG

Condition 1: 94 respondents who are in Intermediate managerial, administrative and professional SEG

Condition 2: 95 respondents who are in Intermediate managerial, administrative and professional SEG

Condition 3: 95 respondents who are in Intermediate managerial, administrative and professional SEG

Condition 1: 115 respondents who are in Supervisory, clerical and junior managerial, administrative and professional SEG

Condition 2: 115 respondents who are in Supervisory, clerical and junior managerial, administrative and professional SEG

Condition 3: 116 respondents who are in Supervisory, clerical and junior managerial, administrative and professional SEG

Condition 1: 82 respondents who are in Skilled manual workers SEG

Condition 2: 82 respondents who are in Skilled manual workers SEG

Condition 3: 83 respondents who are in Skilled manual workers SEG

Condition 1: 61 respondents who are in Semi-skilled and unskilled manual workers SEG

Condition 2: 62 respondents who are in Semi-skilled and unskilled manual workers SEG

Condition 3: 62 respondents who are in Semi-skilled and unskilled manual workers SEG

Condition 1: 41 respondents who are in State pensioners, casual and lowest grade workers, unemployed with state benefits only SEG

Condition 2: 41 respondents who are in State pensioners, casual and lowest grade workers, unemployed with state benefits only SEG

Condition 3: 42 respondents who are in State pensioners, casual and lowest grade workers, unemployed with state benefits only SEG]

[Information sheet presented]

* Caveat - next page:

*Title* Bold and larger text in middle of the page* Please read this information carefully.

[Tick box below each of the following statements saying 'I confirm this is correct'. Respondents must select for all statements before they can proceed OR select 'Prefer not to take part' which will End them]

I agree that the research project has been explained to me to my satisfaction and I agree to take part in this study

I understand that my participation is completely voluntary

I understand that I can withdraw from the survey at any time without giving a reason

I understand that my personal data will be processed for the purposes of this research

I understand that my information will be treated as strictly confidential and handled in accordance with GDPR privacy policy

I understand that the data gathered in the survey will be stored securely and it will not be possible to identify me in any outputs from this research

I confirm that I am at least 18 years old

I confirm that I am the parent or main caregiver of a 6-23-month-old

Separate opt out:

Prefer not to take part [End]

*Caveat to show on the following page:

You will now be asked some questions about baby foods. Please look carefully at the products before answering the questions. There are no right or wrong answers, just go with what you think is best.

If your child has dietary requirements, please assume all the food products you see in this survey meet these dietary requirements [Hide this sentence if ‘None’ is selected at Qxi]

*Note: For Q1-Q5, replace the word ‘baby’ with ‘child’ in all question text for parents of children aged 12-23m

Q1 Product:

Fruit pouch (6m+ label)

| IMAGE DISPLAYED | |
| --- | --- |
| Condition 1: | Condition 2: |
| 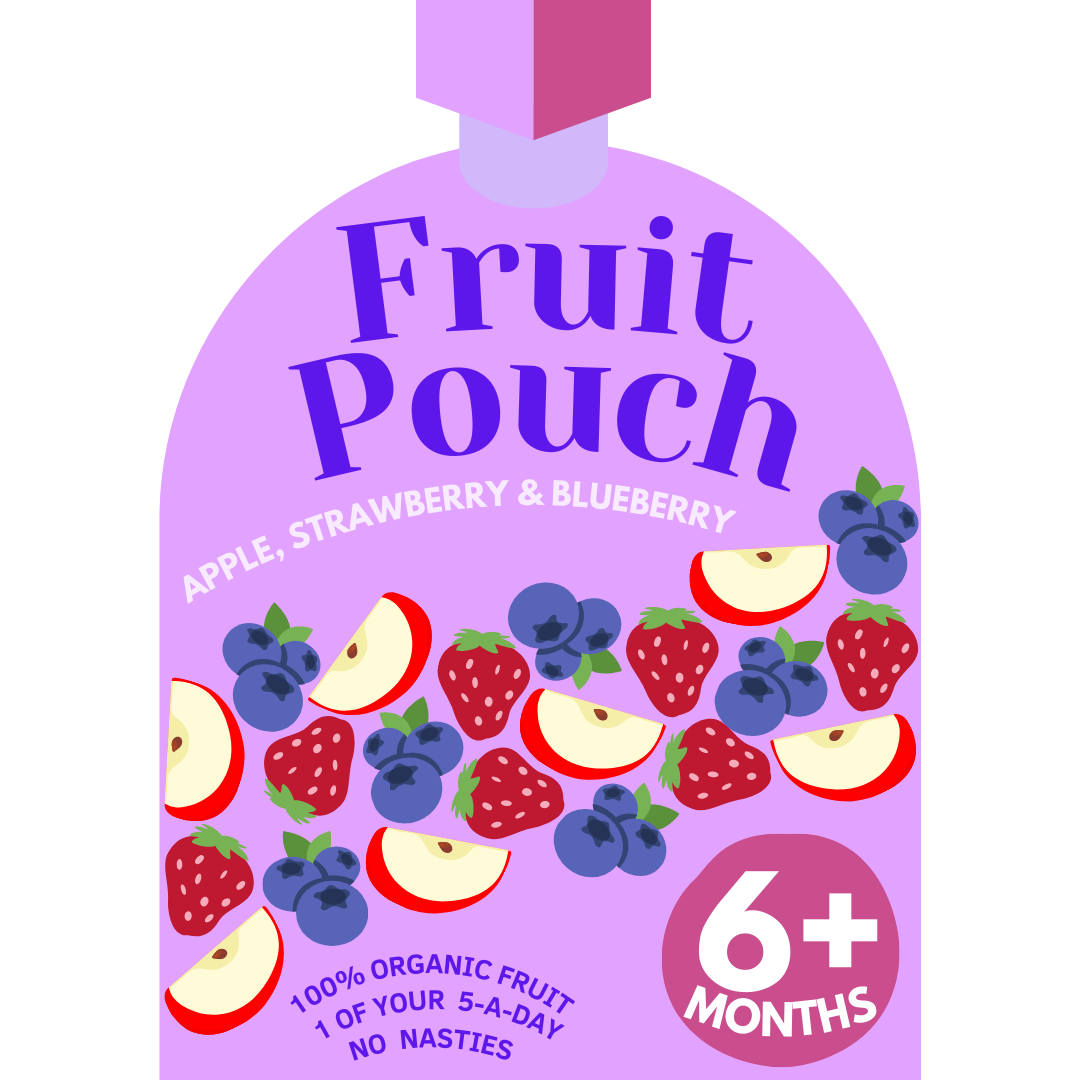 | 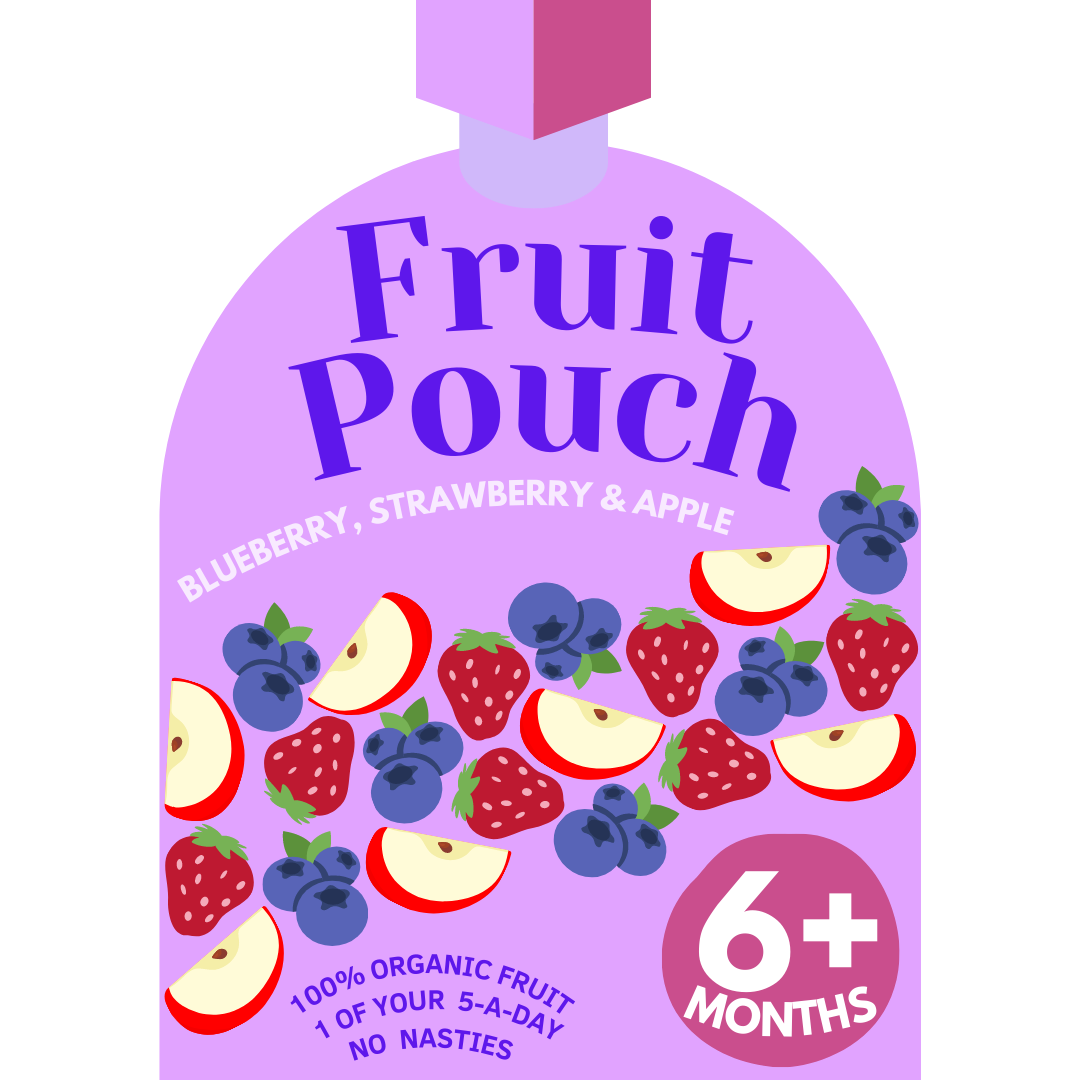 |

*Q1 Conditions:

Apple, strawberries & blueberries [IMAGE CODE Q1C1]

Blueberries, strawberries & apple [IMAGE CODE Q1C2]

*Caveat: Please look at the product below.

Q1.1 Which food would you expect this product to contain in the largest amount?

Apple

Blueberry

Strawberry

All equal

Q1.2 To what extent do you disagree or agree with this statement?

Overall, this product is healthy for my baby.

Strongly disagree

Disagree

Slightly disagree

Neither agree nor disagree

Slightly agree

Agree

Strongly agree

Q1.3 To what extent do you disagree or agree with this statement?

I would buy this product for my baby.

Strongly disagree

Disagree

Slightly disagree

Neither agree nor disagree

Slightly agree

Agree

Strongly agree

*Q2 Product:

Fruit wafer (6m+ label)

| IMAGE DISPLAYED | |
| --- | --- |
| Condition 1: | Condition 2: |
| 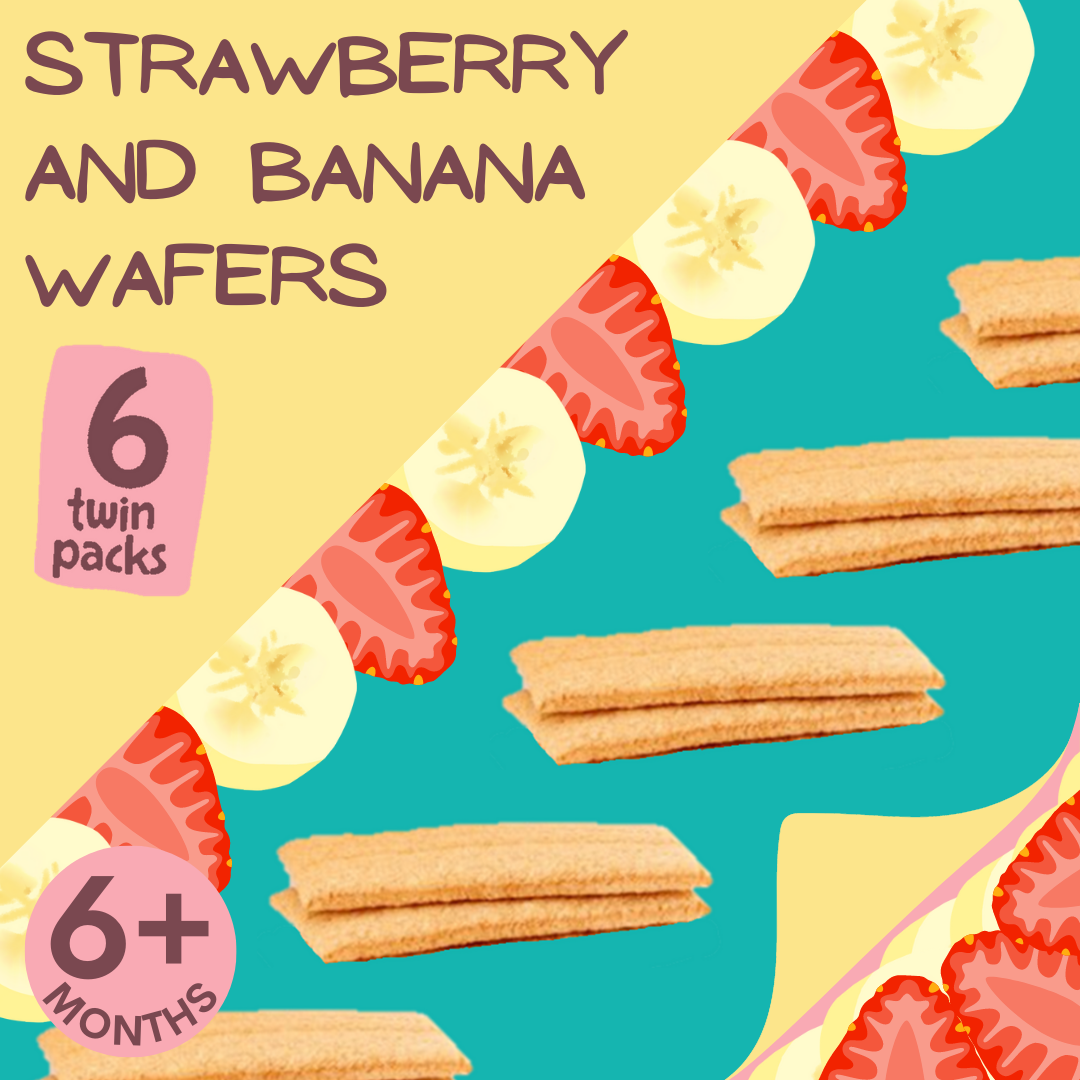 | 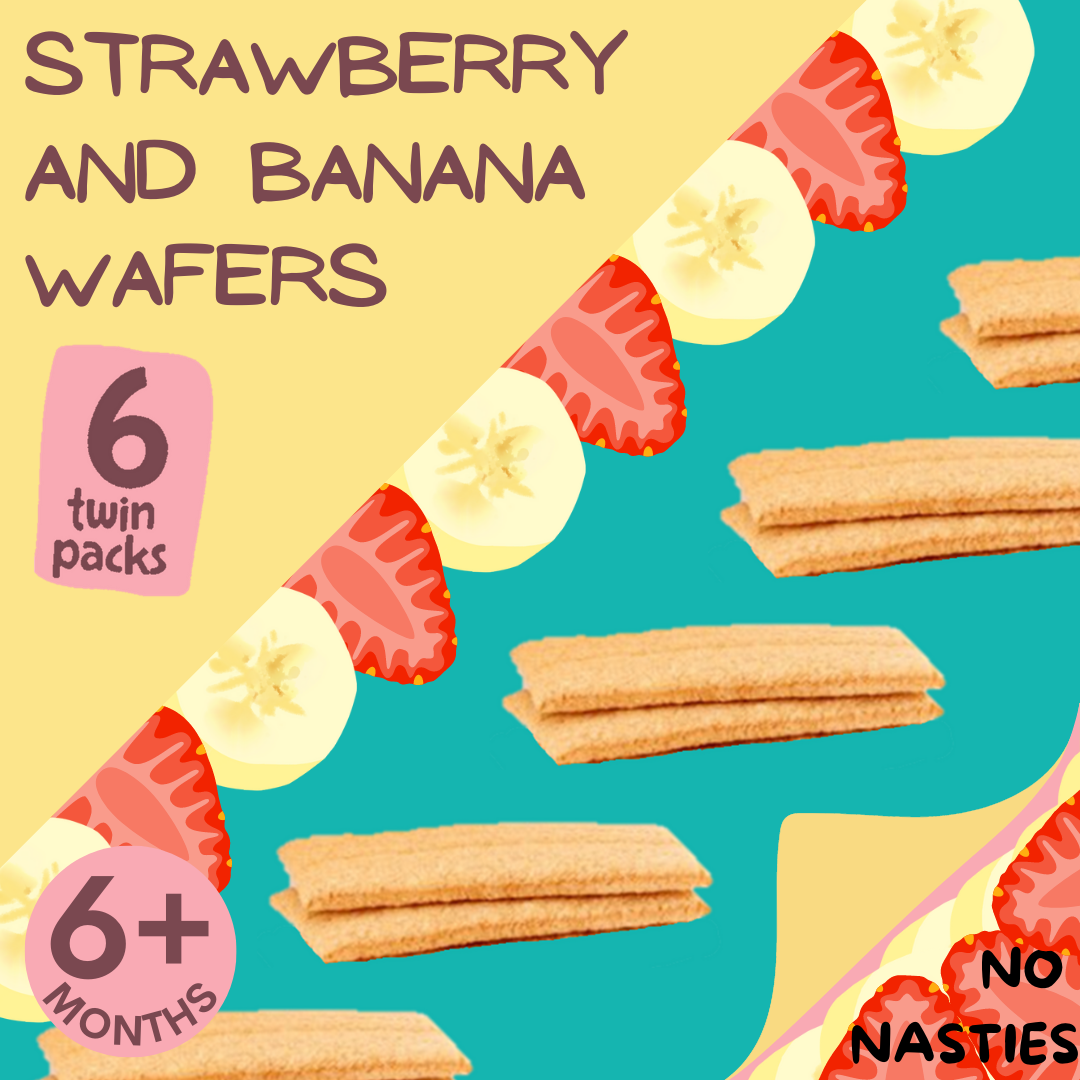 |

*Q2 Conditions:

No marketing messages [IMAGE CODE Q2A1]

‘No Nasties’ [IMAGE CODE Q2A2]

*Caveat: Please look at the product below.

Q2.1 To what extent do you disagree or agree with this statement?

Overall, this product is healthy for my baby.

Strongly disagree

Disagree

Slightly disagree

Neither agree nor disagree

Slightly agree

Agree

Strongly agree

Q2.2. To what extent do you disagree or agree with this statement?

I would buy this product for my baby.

Strongly disagree

Disagree

Slightly disagree

Neither agree nor disagree

Slightly agree

Agree

Strongly agree

*Q3 Product:

Strawberry Biscotti (6m+ label)

| IMAGE DISPLAYED | |
| --- | --- |
| Condition 1: | Condition 2: |
| 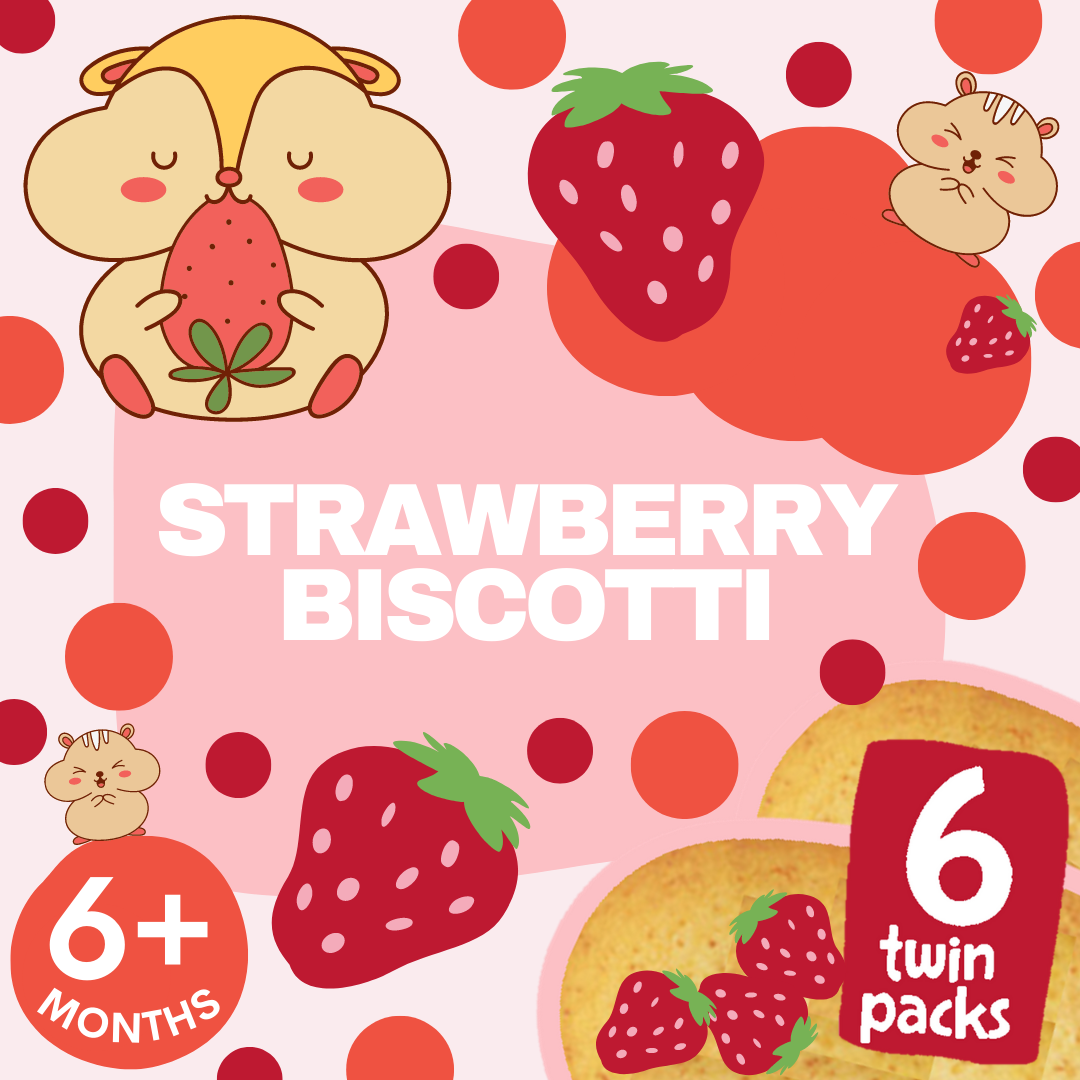 | 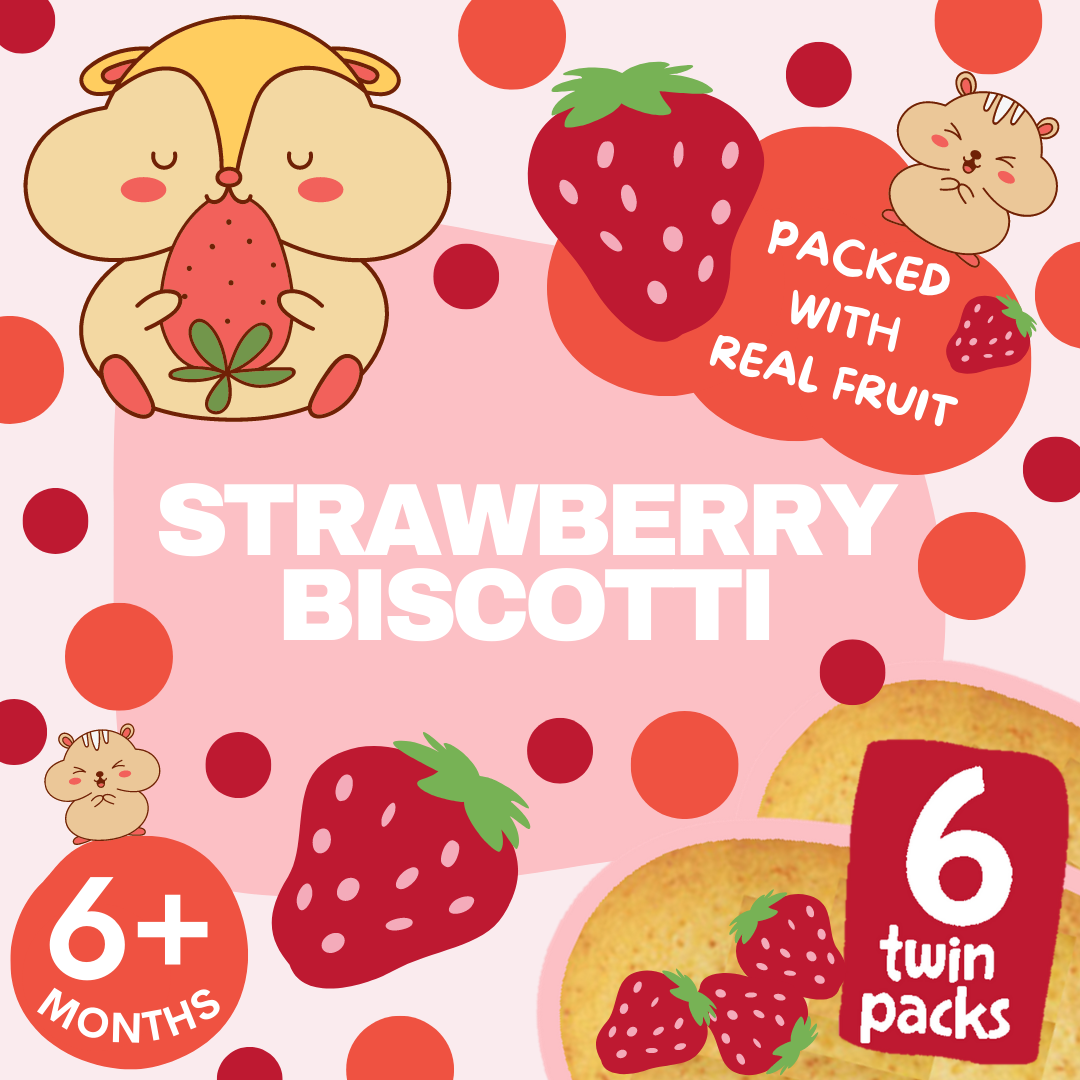 |

*Q3 Conditions:

No marketing messages [IMAGE CODE Q2D1]

‘Packed with real fruit’ [IMAGE CODE Q2D2]

*Caveat: Please look at the product below.

Q3.1 To what extent do you disagree or agree with this statement?

Overall, this product is healthy for my baby.

Strongly disagree

Disagree

Slightly disagree

Neither agree nor disagree

Slightly agree

Agree

Strongly agree

Q3.2 To what extent do you disagree or agree with this statement?

I would buy this product for my baby.

Strongly disagree

Disagree

Slightly disagree

Neither agree nor disagree

Slightly agree

Agree

Strongly agree

*Q4 Product:

Fruity Melty Sticks (6m+ label)

| IMAGE DISPLAYED | |
| --- | --- |
| Condition 1: | Condition 2: |
| 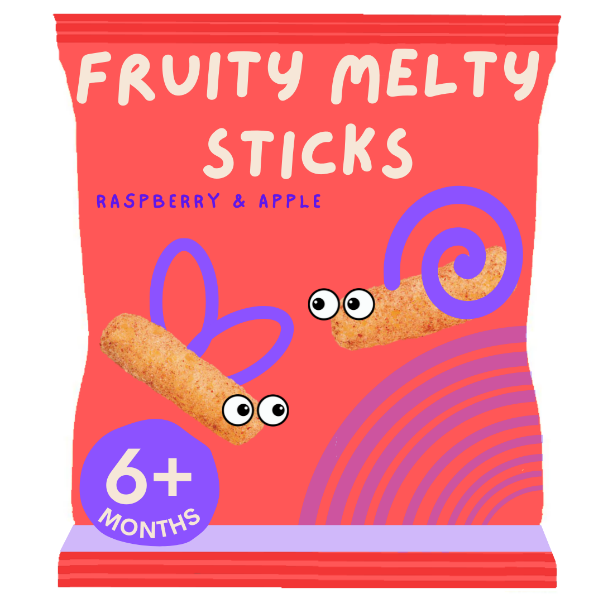 | 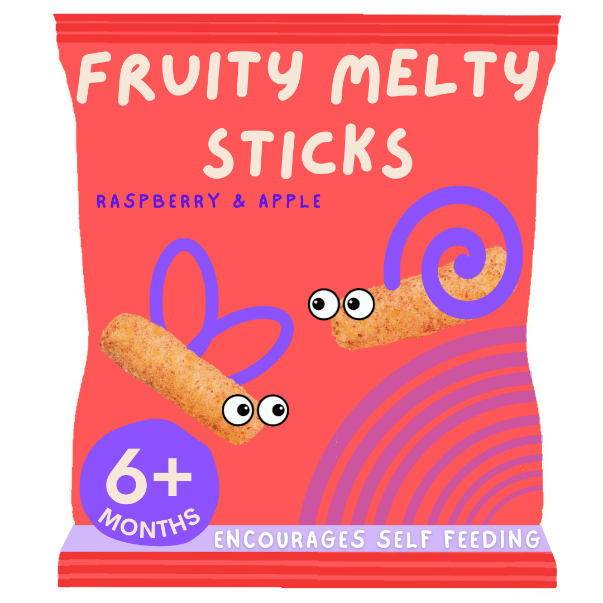 |

*Q4 Conditions:

No marketing messages [IMAGE CODE Q2B1]

Encourages self feeding [IMAGE CODE Q2B2]

*Caveat: Please look at the product below.

Q4.1 To what extent do you disagree or agree with this statement?

Overall, this product is healthy for my baby.

Strongly disagree

Disagree

Slightly disagree

Neither agree nor disagree

Slightly agree

Agree

Strongly agree

Q4.2 To what extent do you disagree or agree with this statement?

I would buy this product for my baby.

Strongly disagree

Disagree

Slightly disagree

Neither agree nor disagree

Slightly agree

Agree

Strongly agree

*Q5 Product:

Apple and Raspberry rice cakes (6m+ label)

| IMAGE DISPLAYED | |
| --- | --- |
| Condition 1: | Condition 2: |
| 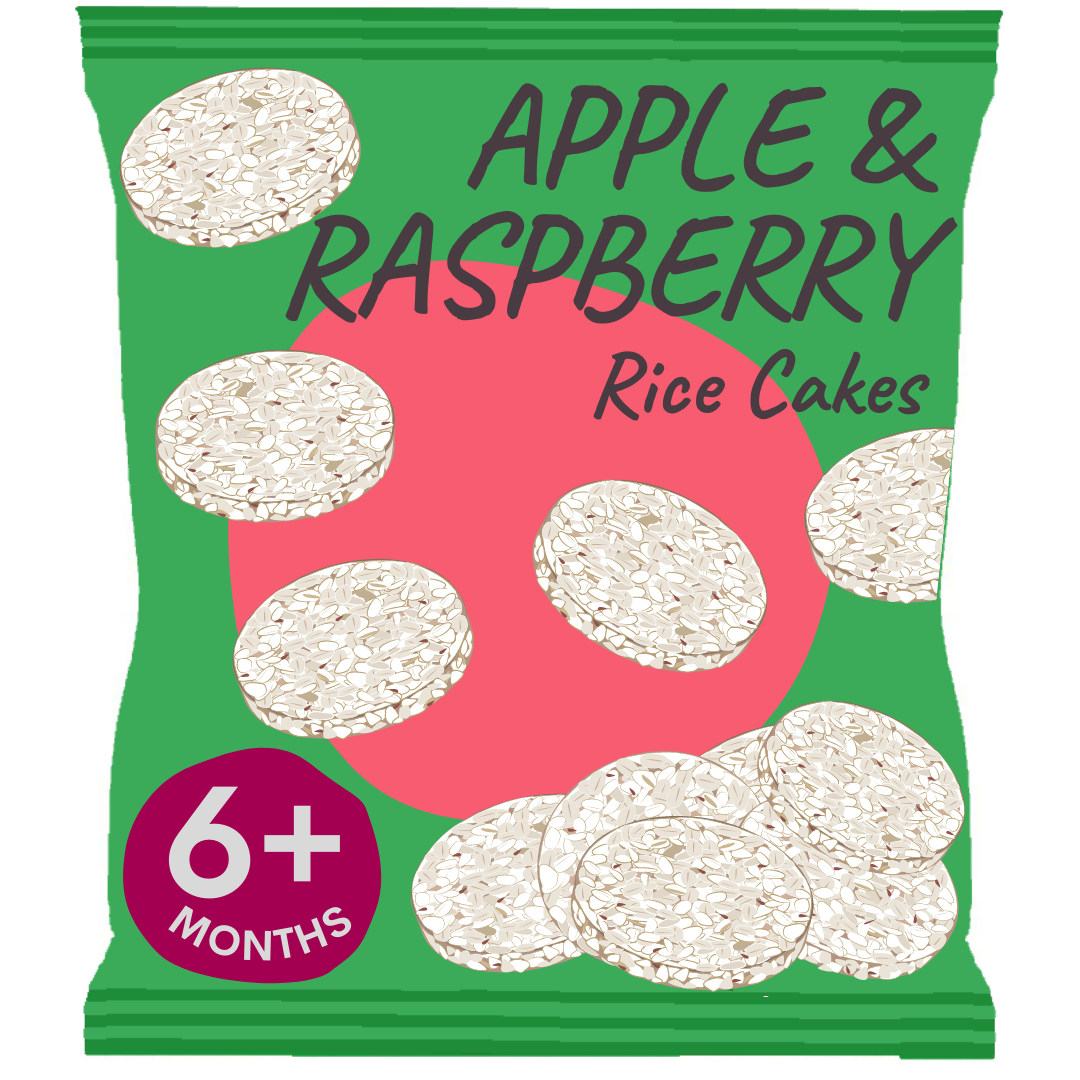 | 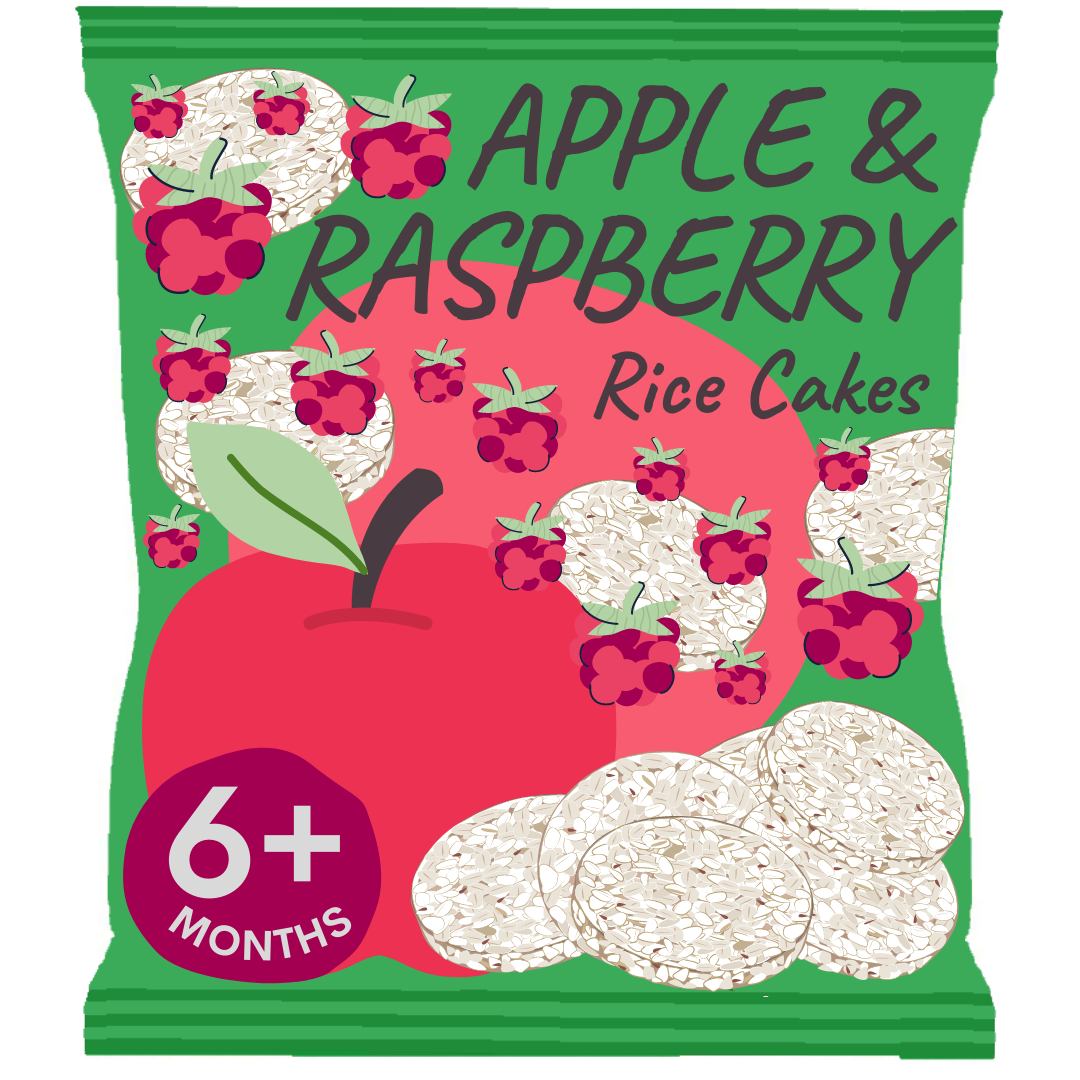 |

*Q5 Conditions:

No fruit images [IMAGE CODE Q2C1]

Fruit image [IMAGE CODE Q2C2]

*Caveat: Please look at the product below.

Q5.1 To what extent do you disagree or agree with this statement?

Overall, this product is healthy for my baby.

Strongly disagree

Disagree

Slightly disagree

Neither agree nor disagree

Slightly agree

Agree

Strongly agree

Q5.2 To what extent do you disagree or agree with this statement?

I would buy this product for my baby.

Strongly disagree

Disagree

Slightly disagree

Neither agree nor disagree

Slightly agree

Agree

Strongly agree

*Q6 conditions:

No sugar messages [IMAGE CODES Q3De1A / Q3De2A / Q3De3A & QNone image]

High sugar indicator [IMAGE CODES Q3De1B / Q3De2B / Q3De3B & QNone image]

High sugar indicator and ‘Contains natural sugars’ [IMAGE CODES Q3De1C / Q3De2C / Q3De3C & QNone image]

*Q6 note: replace the word ‘baby’ with ‘child’ in the question text for parents of children aged 12-23m

Q6.1. Which one of these desserts/puddings would you choose for your baby?

Bread and banana pudding

Totally tropical rice pudding

Very Berry crumble (blueberry, straw, cherry)

None of these

[See Q6.2 if 'None of these' is selected at Q6.1]

Q6.2. What, if anything, would you give your baby/toddler for pudding instead?

Fresh fruit

Dried fruit (e.g. raisins)

Biscuit or sweets (e.g. Jammie Dodger, chocolate buttons)

Other (please give as much detail as possible, including brand)

I wouldn’t give a pudding

*Q7 note: Remove ‘Imagine your baby is older’ text in all question text for parents of children aged 12-23m

*Q7 conditions:

No sugar messages [IMAGE CODES Q3Sn1A / Q3Sn2A / Q3Sn3A & QNone image]

High sugar indicator [IMAGE CODES Q3Sn1B / Q3Sn2B / Q3Sn3B & QNone image]

High sugar indicator and ‘Contains natural sugars’ [IMAGE CODES Q3Sn1C / Q3Sn2C / Q3Sn3C & QNone image]

*Q7 caveat

Q7.1. message for parents of <12mo only: For this question, please imagine your baby is older.

It is 3pm and you are in the supermarket. Your 1- year-old is hungry, but you don’t have anything with you. Which one of these do you pick as a snack?

Banana biscotti

Oat and raisin flapjack bites

Peach & mango fruit rolls

None of these

[See Q7.2 if 'None of these' is selected at Q7.1]

Q7.2. What, if anything, would you give your 1-year-old toddler as a snack instead? Select best match

Fresh fruit or vegetables (e.g. banana, carrot sticks)

Dried fruit (e.g. raisins)

Savoury baby snack (e.g. Veggie puffs)

Savoury snack (e.g. Pom-bear, Wotsit)

Biscuit or sweets (e.g. Jammie Dodger, chocolate buttons)

Other, please specify

I wouldn’t give a snack

*Q8.1 note: replace the word ‘baby’ with ‘child’ in the question text for parents of children aged 12-23m.

*Q8 conditions:

4m+ [IMAGE CODES Q4Me1A / Q4Me2A / Q4Me3A & QNone image]

6m+ [IMAGE CODES Q4Me1B / Q4Me2B / Q4Me3B & QNone image]

Q8.1 For this question, please think back to when your baby was 5-months-old. Which of these foods would you choose for them? Select best match

Butternut, broccoli & carrot jar

Baby rice box

Pumpkin, carrot & potato pouch

None of these

[See Q8.2 if 'None of these' is selected at Q8.1]

Q8.2. What, if anything, would you feed your 5-month old baby instead?

A different brand/type of baby food

Homemade baby food

Only milk (breast or formula)

Other (please specify)

*Q9 note:

Add 'Please imagine your child is older' before the question text for parents of children aged 8-9 months.

Add 'Please imagine your baby is older' before the question text for parents of children aged 6-7 month.

Add ‘Please think back to when your child was younger’ before the question text for parents of children aged 10-23m.

*Q9 conditions:

4m+ [IMAGE CODES Q4Sn1A / Q4Sn2A / Q4Sn3A & QNone image]

6m+ [IMAGE CODES Q4Sn1B / Q4Sn2B / Q4Sn3B & QNone image]

Q9.1. For this question, imagine it is 3pm and you are out with your 9-month-old baby who is hungry. You don’t have any food with you. Which of these would you choose for your baby?

Strawberry and banana rice cakes

Apple wafer

Melty veggie sticks

None of these

[See Q9.2 if 'None of these' is selected at Q9.1]

Q9.2. What would you give your 9-month-old baby as a snack instead?

A snack labelled ‘12m+’

Fresh fruit (e.g. banana)

Dried fruit (e.g. raisins)

Another snack (e.g. Jammie Dodger, Wotsits)

Milk (breast or formula)

Nothing

Other (please specify)

*Q10 note:

Add 'Please imagine your child is older' before the question text for parents of children aged 8-9 months.

Add 'Please imagine your baby is older' before the question text for parents of children aged 6-7 month.

Add ‘Please think back to when your child was younger’ before the question text for parents of children aged 10-23m.

*Q10 conditions:

4m+ [IMAGE CODES Q4Dr1A / Q4Dr2A / Q4Dr3A & QNone image]

6m+ [IMAGE CODES Q4Dr1B / Q4Dr2B / Q4Dr3B & QNone image]

Q10.1. For this question, imagine it is a hot day and you are out with your 9-month-old baby who is thirsty. Which of these would you choose for your baby?

pear & peach

apple & blueberry

apple

None of these

[See Q10.2 if 'None of these' is selected at Q10.1]

Q10.2. What, if anything, would you give your 9-month-old baby to drink instead?

Water

Milk (breast or formula)

Another drink (e.g. Innocent smoothie, Fruit Shoot)

Other (please specify)

Q11 caveat for parents of children aged 6-11 months: For the next set of questions please think about how you feed your baby now.

Q11 caveat for parents of children aged 12-23 months: For the next set of questions please think about how you feed your child now.

*Q11-Q14 note: replace the word ‘baby’ with ‘child’ for parents of children aged 12-23m

Q11.1. To what extent do you disagree or agree with this statement?

If my baby was crying and I needed to make a phone call, I would give them a snack to keep them quiet.

Strongly disagree

Disagree

Slightly disagree

Neither agree nor disagree

Slightly agree

Agree

Strongly agree

Q11.2. When choosing a snack for your baby how important are the following:

*Matrix*

Rows:

Price

Brand reputation

My child asks for it

Appearance of pack e.g. colour, pictures

How messy my baby will get

Good for my baby’s health

Good for my baby’s development

Keeps my baby busy

My baby can feed it to themselves

Conveniently packaged/no preparation

Labelled as suitable for their age

Soft or melty texture

No nasties/additives

No added sugar

Contains fruit/vegetables

Natural ingredients

Organic

Other

Columns: 1 Not at all important - 10 Very important

[See Q11.3 if ‘2’ or above is selected for 'Other' at Q11.2]

Q11.3. You said that something other than the listed options on the previous question was important when choosing a snack for your baby, please expand on this below:

[Open text]

Q11.4. How often, if at all, do you encourage your baby to eat more?

Always

Often

Sometimes

Rarely

Never

Q11.5. How often, if at all, do you give your baby a cup of water with meals?

Always

Often

Sometimes

Rarely

Never

Q11.6. To what extent do you disagree or agree with this statement? ‘My baby shows obvious signs when they have had enough to eat’

Strongly disagree

Disagree

Slightly disagree

Neither agree nor disagree

Slightly agree

Agree

Strongly agree

Q11.7. To what extent do you disagree or agree with this statement? ‘I stop feeding my baby straight away if they show signs that they’ve had enough to eat (e.g. turning their head away).’

Strongly disagree

Disagree

Slightly disagree

Neither agree nor disagree

Slightly agree

Agree

Strongly agree

Q11.8. How long, if at all, did you breastfeed your baby for (please include mixed feeding and answer to the nearest month)?

Currently breastfeeding

Didn’t breastfeed at all

Breastfed until baby was 1 month

Breastfed until baby was 2 months

Breastfed until baby was 3 months

Breastfed until baby was 4 months

Breastfed until baby was 5 months

Breastfed until baby was 6 months

Breastfed until baby was 7 months

Breastfed until baby was 8 months [Hide if '8-9', '10-11', '12-14', '15-16', '17-18', '19-21' or '22-23' is not selected in Qviii]

Breastfed until baby was 9 months [Hide if '8-9', '10-11', '12-14', '15-16', '17-18', '19-21' or '22-23' is not selected in Qviii]

Breastfed until baby was 10 months [Hide if '10-11', '12-14', '15-16', '17-18', '19-21' or '22-23' is not selected in Qviii]

Breastfed until baby was 11 months [Hide if '10-11', '12-14', '15-16', '17-18', '19-21' or '22-23' is not selected in Qviii]

Breastfed until baby was 12 months [Hide if '12-14', '15-16', '17-18', '19-21' or '22-23' is not selected in Qviii]

Breastfed until baby was 13 months [Hide if '12-14', '15-16', '17-18', '19-21' or '22-23' is not selected in Qviii]

Breastfed until baby was 14 months [Hide if '12-14', '15-16', '17-18', '19-21' or '22-23' is not selected in Qviii]

Breastfed until baby was 15 months [Hide if '15-16', '17-18', '19-21' or '22-23' is not selected in Qviii]

Breastfed until baby was 16 months [Hide if '15-16', '17-18', '19-21' or '22-23' is not selected in Qviii]

Breastfed until baby was 17 months [Hide if '17-18', '19-21' or '22-23' is not selected in Qviii]

Breastfed until baby was 18 months [Hide if '17-18', '19-21' or '22-23' is not selected in Qviii]

Breastfed until baby was 19 months [Hide if '19-21' or '22-23' is not selected in Qviii]

Breastfed until baby was 20 months [Hide if '19-21' or '22-23' is not selected in Qviii]

Breastfed until baby was 21 months [Hide if '19-21' or '22-23' is not selected in Qviii]

Breastfed until baby was 22 months [Hide if '22-23' is not selected in Qviii]

Breastfed until baby was 23 months [Hide if '22-23' is not selected in Qviii]

Q12.1. How old was your baby when you started weaning (giving any food other than milk or water)?

My baby only has milk or water [Skip Q12.2, 12.3, 13.1, 13.2 and Q14.1]

Less than 3 months

4 months

5 months

6 months

7 months

8 months [Hide if ‘6-7’ is selected at Qviii]

Older than 8 months [Hide if ‘6-7’ is selected at Qviii]

Q12.2. What was the first food you gave your baby? Please give as much detail as possible, including whether this was homemade or shop bought and if so, which brand.

*Open response*

I don’t remember

Q12.3. How old was your baby when you started giving any snacks between meals?

Less than 3 months

4 months

5 months

6 months

7 months

8 months [Hide if ‘6-7’ is selected at Qviii]

9 months [Hide if ‘6-7’ is selected at Qviii]

10 months [Hide if ‘6-7’ is selected at Qviii]

11 months [Hide if ‘6-7’ is selected at Qviii]

12 months [Hide if ‘6-7’ is selected at Qviii]

Older than 12 months [Hide if ‘6-7’ is selected at Qviii]

*Respondents cannot enter a value lower than that entered at Q12.1*

*Q13-Q14 note: Replace the word ‘baby’ with ‘child’ for parents of children aged 12-23m.

Q13.1 How many times a day on average, if at all, do you give your baby food (including meals and snacks, but not counting milk)?

Less than once a day

[options of 1 – 20]

More than 20 times a day

[See Q13.2 unless 'Less than once a day' is selected at Q13.1]

Q13.2. How many of the times you give your baby food each day would you think of as snacks?

[options of 1 – 20]

More than 20 times a day

*Respondents cannot select a greater value than at Q13.1.

Q13.3. How many milk feeds do you give your baby on a typical day (24 hours)?

Less than once a day

[options of 1 – 20]

More than 20 times a day

Q13.4. How many drinks, other than milk or water, do you give your baby on a typical day?

Less than one a day

[options of 1 – 20]

More than 20 a day

Q14.1 Have you regularly given your baby any of the following shop-bought baby foods (either now or when they were younger): (Tick all that apply)

|  | As part of a meal | As a snack between meals | Neither *Exclusive* |
| --- | --- | --- | --- |
| Shop-bought savoury finger foods for babies (e.g. veggie puffs) |  |  |  |
| Shop-bought fruit finger foods for babies (e.g fruit puffs, wafers, biscuits or oaty bars) |  |  |  |
| Fruit pouches or jars |  |  |  |
| Savoury pouches, jars or trays |  |  |  |

[Skip Q14.2 if 'Neither' selected for all rows at Q14.1 or 'My baby only has milk or water' was selected at Q12.1]

Q14.2. When did you first begin to give your baby the following shop-bought baby foods with a meal *BOLD AND UNDERLINED*?

|  | Less than 4 months | 4-5 months | 6-11 months | 12 months or more [Hide if ‘6-7’ is selected at Qviii] |
| --- | --- | --- | --- | --- |
| [Show options selected ‘With a meal’ at Q14.1] |  |  |  |  |

[Skip Q14.3 if 'Neither' selected for all rows at Q14.1 or 'My baby only has milk or water' was selected at Q12.1]

Q14.3. When did you first begin to give your baby the following shop-bought baby foods as a snack between meals *BOLD AND UNDERLINED*?

|  | Less than 4 months | 4-5 months | 6-11 months | 12 months or more [Hide if ‘6-7’ is selected at Qviii] |
| --- | --- | --- | --- | --- |
| [Show options selected ‘As a snack between meals’ at Q14.1] |  |  |  |  |

Q15.1. What is the recommended age for babies to start weaning (having foods other than milk)?

3 months or younger

4 months

5 months

6 months

7 months

8 months

9 months

10 months or older

I don’t know

Q15.2. What is the recommended age for babies to start having snacks between meals?

5 months or younger

6 months

7 months

8 months

9 months

10 months

11 months

12 months

13 months or older

I don’t know

Q16.1 To what extent do you disagree or agree with this statement?

I think my baby needs snacks between meals. [Show ‘I think my baby needed snacks between meals when they were younger than 12 months’ for parents of children aged 12-23m]

Strongly disagree

Disagree

Slightly disagree

Neither agree nor disagree

Slightly agree

Agree

Strongly agree

Q16.2 To what extent do you disagree or agree with this statement?

If I had been told by my health visitor or doctor not to give snacks to my baby before 12 months I would not have given them snacks.

Strongly disagree

Disagree

Slightly disagree

Neither agree nor disagree

Slightly agree

Agree

Strongly agree

Q16.3 To what extent do you disagree or agree with this statement?

Natural sugar in baby food is not bad for them

Strongly disagree

Disagree

Slightly disagree

Neither agree nor disagree

Slightly agree

Agree

Strongly agree

Q17. To what extent do you disagree or agree that foods and drinks labelled as suitable for babies are…

|  | Strongly disagree | Disagree | Slightly disagree | Neither agree nor disagree | Slightly agree | Agree | Strongly agree |
| --- | --- | --- | --- | --- | --- | --- | --- |
| Low in sugar |  |  |  |  |  |  |  |
| Low in salt |  |  |  |  |  |  |  |
| Don’t have added sugar |  |  |  |  |  |  |  |
| Don’t have added salt |  |  |  |  |  |  |  |

Q18. Have you received information about feeding your baby from any of the following and if so, was it helpful?

|  | No advice seen/received | Not at all helpful | Only slightly helpful | Quite helpful | Very helpful |
| --- | --- | --- | --- | --- | --- |
| Family & friends |  |  |  |  |  |
| Health Visitor |  |  |  |  |  |
| GP/docto |  |  |  |  |  |
| NHS/Start4life website |  |  |  |  |  |
| Baby food company (e.g. Ella’s Kitchen) |  |  |  |  |  |
| Parent’s website (e.g. mumsnet) |  |  |  |  |  |
| Social media groups (e.g. Instagram, Facebook, WhatsApp) |  |  |  |  |  |
| Apps from a food company e.g. Ella’s Kitchen |  |  |  |  |  |
| Apps without adverts e.g. Baby Buddy |  |  |  |  |  |
| Books |  |  |  |  |  |
| Other |  |  |  |  |  |

[See Q18.1 unless 'No advice seen/received' is selected for 'Other' at Q18]

Q18.1. You said that you recieved information about feeding your baby from a source other than those listed in the previous question, please expand on this below:

[Open text]

Q19. Please tell us what about weaning advice or information you found particularly helpful, including the names of any websites, apps, social media groups etc.

Please give as much detail as possible

(Open question)

*Opt outs:

I didn’t find any weaning advice particularly helpful

I haven’t received any weaning advice or information

Q20. Regarding feeding your baby / toddler, what, if anything, would you have liked more advice about? (tick all that apply)

When to start weaning (introducing food or drink other than milk or water)

Which foods to give at different ages

Portion sizes for different ages

How to prepare baby food at home

How to store baby food

Allergies

Baby-led weaning

Other, please specify

I wouldn’t have liked more advice about anything *exclusive*

Q21. What do you think would be the best way to provide feeding advice to new parents? (Please give as much detail as possible about who would be involved, when advice would be given, what the advice might look like etc.)

(Open question)

I don’t know

-------- END OF SURVEY--------

**Interview schedule**

**Photo Elicitation -**3-5 photos of baby/toddler food packaging that you see in the week before your session. Label these photos with your initial thoughts/reactions (e.g. what does it make you think of? What does it make you think about the product?)

**Intro researchers** – facilitator & co-facilitator (notes, photos of foods, timekeeper). Foods coming out (noisy!, rice cakes available). From uni not company.

**Aims:**

1. We want to understand more about how parents view labels on baby foods & how you use them when you’re deciding what to feed your baby.

2. Explore whether making changes to labels would make it easier for you to choose healthy foods.

**Before we start – run through:**

1. Only one person speaks at a time (recording) & try to give everyone an equal chance to speak. We want a range of views, say your thoughts at any point (ignore recorder) do talk to each other – I’m here to guide the conversation.

2. Confidentiality – please respect the privacy of others in the group by not repeating what is discussed here outside the group. (Recording – remove names/places anything that could identify).

3. If need to get up for baby, no problem, bring baby to table and come back asap.

Check all agree? Any questions? 1 hour ok? Recording ok?

1. Each person can say their first name and the age/s of child/children. When we start recording, I’ll ask everyone to say their name in order around the table for the transcriber & 2 foods beginning with the same letter & same again about ½ way through – help transcriber know who was speaking.

2. Something you craved when you were pregnant, or partner or your favourite food? Cat/dog? Chocolate or crisps?

**[Start recording]**

**1. Say your name (2 mins)** followed by 2 foods beginning with the same letter

**2. Photo elicitation task (5 mins)** (all photos on table)

Here are some of the images that caught peoples eye this week. Thanks. Please find an image now that you feel you would pick up for a closer look if you saw it in supermarket. What is it that drew you to it? Just initial thoughts – colours/bought before/brand/new to you

**3. Product Mapping Exercise (15 mins)**

On the table you can see a range of food products that people give their babies & toddlers - products from different companies, different types of foods and flavours. **TASK:** What I’d like you to do as a group is to put them into 2 groups. 1. Everyday foods & 2. Occasional foods/treats (may or may not be the same size). And please think aloud/discuss while you’re doing this.

What your initial thoughts and impression of the foods so, You have 2 minutes to do this, Go!

Now I’d like you to talk through what helped you decide (organic, pictures, brand). Would anyone like to move anything? Why?

**[repeat names and foods]**

**4. Addition of sugar flag to labels (20 mins)**

Next, I’d like to think about the amount of sugar in foods. Is sugar something you feel you need to think about when you’re choosing baby food?

**TASK**: I’d like you to put any foods that you think are high in sugar into a separate group – 2 mins! What was it about these that give the impression they’re high/low?

An idea has been suggested – that baby foods that have high levels of sugar should have a label on them to help parents. If this suggestion was followed, then these foods would get a label on them.

Can you tell me how you feel about that? (surprised/expected/disbelief)

How would you feel about these labels being added? How would it affect your choices? What are your thoughts about the black label? (DHSC)?

**5. Discussion of current guidelines (15 Mins)**

This is an image from the NHS website about weaning (read out) – what do you understand be this? (Had you heard this? Does this match up with what you’ve read/been advised/seen on labels/your experience? where did you get weaning advice?)

This is another image – from the NHS about snacks (read out). – what do you understand be this? meaning of ‘snacks’? (Had you heard this? How does this match up with what you’ve read/been advised/seen on labels? do <12m need snacks?

This is the last image – from the NHS website about sugar (read out) - what do you understand by this? (Had you heard this? How does this match up with what you’ve heard? How easy is it to follow? Would label changes help?

**6. Last thoughts (3 mins)** – If you were in charge of baby food labelling regulations – Would you change anything to make it easier for parents to choose healthy foods, and if so what would it be? Anything else that would help you choose healthy foods for your baby?

**[Stop recording]**

Thanks, good discussion, appreciate sharing thoughts, request don’t share discussion. If anyone’s feeling maybe should have done things differently – it’s so confusing & we can only do our best, your contribution today helps us towards making things a bit simpler & easier for everyone.
